# USP7-Dependent Regulation of TRAF Activation and Signaling by a Viral Interferon Regulatory Factor Homologue

**DOI:** 10.1128/JVI.01553-19

**Published:** 2020-01-06

**Authors:** Qiwang Xiang, Hyunwoo Ju, John Nicholas

**Affiliations:** a Sidney Kimmel Comprehensive Cancer Center at Johns Hopkins, Department of Oncology, Johns Hopkins University School of Medicine, Baltimore, Maryland, USA; Northwestern University

**Keywords:** viral interferon regulatory factor-2, ubiquitin-specific protease 7, herpesvirus-associated ubiquitin-specific protease, human herpesvirus 8, replication, latency, tumor necrosis factor receptor-associated factors, TRAF3, TRAF6

## Abstract

Human herpesvirus 8 (HHV-8) encodes four viral interferon regulatory factors (vIRFs 1 to 4), all of which are expressed during lytic replication and inhibit a variety of antiviral signaling pathways. Viral IRFs 1, 2, and 3 are also expressed during latency in primary effusion lymphoma (PEL) cells, and vIRF-1 and vIRF-3 have been reported to promote PEL cell viability. Viral IRFs 1, 3, and 4 are known to interact with ubiquitin-specific protease 7 (USP7); interactions of vIRF-1 and vIRF-3 with USP7 promote PEL cell viability and regulate productive replication. Here, we report that vIRF-2 also targets USP7, utilizing a PSTS motif matching the USP7 N-terminal domain-binding A/PxxS consensus, but uniquely requires catalytic domain residues for intracellular interaction. In functional and mechanistic analyses, tumor necrosis factor receptor-associated factor (TRAF)-mediated signaling and associated polyubiquitination of TRAFs 3 and 6, specifically, were regulated negatively by USP7 and positively by vIRF-2-USP7 interaction, the latter competing for USP7-TRAF association. Using depletion, depletion-complementation, and targeted mutagenesis approaches, vIRF-2 was determined to promote latent PEL cell viability, likely independently of USP7 interaction, while lytic replication was inhibited by vIRF-2, in part or in whole via USP7 interaction. Together, our data identify a new molecular determinant of USP7 recognition, TRAF3/6-specific targeting by the deubiquitinase, associated activation of these TRAFs by vIRF-2, and activities of vIRF-2 and vIRF-2-USP7 interaction in HHV-8 latent and lytic biology.

**IMPORTANCE** Human herpesvirus 8-encoded IRF homologues were the first to be identified in a virus. Through inhibitory interactions with cellular IRFs and other mediators of antiviral signaling, the vIRFs are believed to be essential for productive replication and also for latency in particular cell types. The deubiquitinase USP7 is a regulator of key cellular pathways, modulates HHV-8 latent and lytic infection, and is targeted by vIRFs 1, 3, and 4. Here, we report that vIRF-2 also interacts with USP7, via a means distinguishable from USP7 interactions with other vIRFs and other proteins, that this interaction modulates antiviral signaling via disruption of USP7 interactions with innate immune signaling proteins TRAF3 and TRAF6, and that vIRF-2 targeting of USP7 regulates HHV-8 productive replication. The presented data are the first to identify vIRF-2 targeting of USP7 and its role in HHV-8 biology, expanding our understanding of the repertoire and importance of virus-host interactions.

## Abstract (precis)

Human herpesvirus 8-encoded IRF homologues were the first to be identified in a virus. Through inhibitory interactions with cellular IRFs and other mediators of antiviral signaling, the vIRFs are believed to be essential for productive replication and also for latency in particular cell types. The deubiquitinase USP7 is a regulator of key cellular pathways, modulates HHV-8 latent and lytic infection, and is targeted by vIRFs 1, 3, and 4. Here, we report that vIRF-2 also interacts with USP7, via a means distinguishable from USP7 interactions with other vIRFs and other proteins, that this interaction modulates antiviral signaling via disruption of USP7 interactions with innate immune signaling proteins TRAF3 and TRAF6, and that vIRF-2 targeting of USP7 regulates HHV-8 productive replication. The presented data are the first to identify vIRF-2 targeting of USP7 and its role in HHV-8 biology, expanding our understanding of the repertoire and importance of virus-host interactions.

## INTRODUCTION

Human herpesvirus 8 (HHV-8) is an oncogenic virus associated with the endothelial tumor Kaposi’s sarcoma (KS), the B-cell disorders primary effusion lymphoma (PEL) and multicentric Castleman’s disease (MCD), and MCD-related KICS (KS-associated herpesvirus-induced cytokine syndrome) (1, 2). These diseases involve latently infected transformed and/or hyperplastic cells, but it is also believed that viral and cellular cytokines and other factors secreted from lytically infected cells are involved critically in disease onset and progression (3–5). The four viral interferon regulatory factors encoded by HHV-8 are believed to play important roles not only in viral latency and productive replication, in general through suppression of stress-related pathways (6, 7), but also in disease pathogenesis. For example, shortly after its discovery, viral interferon regulatory factor 1 (vIRF-1) was reported to function as an oncogene in experimental systems (8), and its subsequently reported inhibitory interactions with the tumor suppressor p53 and p53-activating ATM kinase provided a possible mechanistic explanation for such activity (9–11). It is notable that vIRF-3 also interacts with and inhibits p53 (12) and that vIRF-4 interacts with and stabilizes MDM2, an E3 ubiquitin ligase and destabilizer of p53 (13). However, HHV-8 vIRFs have since been discovered to interact with a variety of cellular factors involved in innate immunity, stress signaling, and apoptosis (6, 7, 14), and through these interactions they are likely to promote not only latently infected cell viability, when expressed in these cells, but also productive replication. It is notable, for example, that vIRF-1 targets and inhibits mitochondrial antiviral signaling (MAVS) protein and stress-induced and -activated BH3-only proteins Bim and Bid, and these interactions, specifically, have been shown to contribute to the proreplication activities of vIRF-1 (15–17). Of relevance in this respect are the reported interactions of vIRF-1, vIRF-2, and vIRF-3 with cellular IRFs and/or IRF-interacting transcriptional coactivators p300 and CBP, required for antiviral interferon gene induction, indicating that these vIRFs mediate proreplication activities through suppression of interferon signaling (18–23). Other likely mechanisms of vIRF proviral activities include inhibitory interactions of vIRF-2 with double-stranded RNA-activated kinase (effecting antiviral responses and apoptosis) (24) and inhibitory targeting by vIRF-3 of the NF-κB-activating and inflammatory cytokine-inducing IκB kinase β (IKKβ) component of the IKK complex (25).

Ubiquitin-specific protease 7 (USP7), alternatively called herpesvirus-associated USP (HAUSP), is known to interact with vIRF-1, vIRF-3, and vIRF-4 (26–28), and here we report that vIRF-2 also targets the deubiquitinase. Interactions of vIRF-1 and vIRF-3 are through one and a pair, respectively, of EGPS motifs, which bind to the N-terminal domain (NTD), specifically TRAF-like domain sequences, of USP7 (26, 28); vIRF-4 has an equivalent motif (ASTS) matching the A/PxxS consensus motif found in most USP7-interacting proteins but also possesses an adjacent sequence that interacts with the catalytic domain of USP7 (28). USP7 is a known deubiquitinase of both p53 and its E3 ubiquitin ligase MDM2, stabilizing both substrates via removal of K48-linked polyubiquitin adducts. When p53- and MDM2-targeting ATM kinase is inactive, the affinity of USP7 for MDM2 is higher than it is for p53; under conditions of DNA damage response activation, however, the reverse is true and p53 is stabilized by ATM phosphorylation of MDM2 (and associated MDMX/MDM4) and consequent reduced USP7 binding to the p53 regulator (29). The interactions of all HHV-8 vIRFs with USP7 suggest that p53 regulation, and the resulting apoptotic inhibition, is a common node of vIRF activity in the context of infection. Indeed, it has been reported that vIRF-3 depletion in PEL cells leads to increased levels of p53 (30). On the other hand, our own studies detected no changes in p53 levels upon depletion of vIRF-3 or vIRF-1 in the context of latently infected or lytically reactivated PEL cells, while phenotypic effects of such depletions on latent PEL cell viability and productive replication were evident (27). Equivalent phenotypes, absent p53 changes, were evident also in lytically reactivated HHV-8^+^ iSLK epithelial cells infected with wild-type (WT) or vIRF-1- or vIRF-3-null viruses. The detected vIRF-associated phenotypes were mediated, at least in part, through vIRF-1 and vIRF-3 interactions with USP7 (27). Furthermore, direct depletion of USP7, while not altering p53 levels, promoted apoptosis and inhibited productive replication in PEL cells (27), indicating the overall importance of USP7 activity in HHV-8 biology. This had previously been inferred in PEL cells through the delivery of vIRF-4-based USP7-interacting peptides, which effectively blocked USP7 enzymatic activity and induced p53-dependent cell death (28). Thus, while p53 regulation is clearly one mechanism by which vIRF-USP7 interactions could affect HHV-8 biology, it is apparent that there are other means by which these interactions can exert biological effects, presumably through additional USP7 substrates. In this respect, it is notable that numerous USP7-interacting proteins have been reported, and some of these are known to be substrates of the deubiquitinase. Notably, among reported USP7-interacting proteins are several E3 ubiquitin ligases, in addition to p53-targeting MDM2, and these include signal-transducing tumor necrosis factor (TNF) receptor-associated factors (TRAFs) 1 to 6 (31–33); TRAF6 is the only TRAF so-far reported to be a substrate of USP7, and this is relevant to the inhibition of antiviral signaling by herpes simplex virus immediate-early protein ICP0 (34). It has not been determined how reported vIRF-1, vIRF-3, or vIRF-4 interactions with USP7 affect deubiquitinase- or independently mediated activities of USP7. Other HHV-8 proteins known to interact with USP7 are viral latency-associated nuclear antigen (LANA) and the ORF45 immediate-early and tegument-contained protein encoded by ORF45 (pORF45). USP7 targeting by LANA appears to inhibit DNA replication (35), a phenomenon mirroring the situation reported for the functionally equivalent EBNA1 latency protein of Epstein-Barr virus (36). One consequence of USP7 targeting by pORF45 is the stabilization of another tegument protein, specified by ORF33, through complexing and effective recruitment of USP7 activity to pORF33, resulting in protease-mediated removal of K48-linked polyubiquitin adducts and thereby rescue from proteasomal degradation (37).

Here, we identify the targeting of USP7 by HHV-8 vIRF-2, characterize the unique requirements for this interaction, identify functionally significant and vIRF-2-regulated interactions of USP7 with TRAFs 3 and 6, and reveal the significance of vIRF-2 to promotion of latently infected PEL cell viability and of vIRF-2-USP7 interaction to virus productive replication. Beyond revealing USP7 as a common target of all HHV-8 vIRFs, this study identifies specific and unexpected activities and underlying mechanisms associated with vIRF-2 interaction with the deubiquitinase.

## RESULTS

### HHV-8 vIRF-2 interacts with USP7.

**FIG 1:**
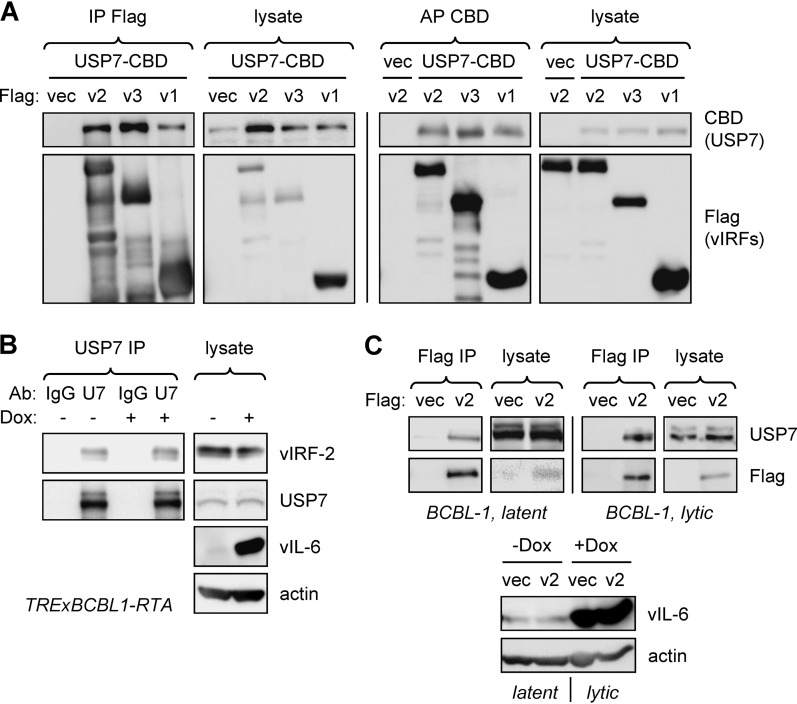
Interaction of vIRF-2 with USP7. (A) HEK293T cells were cotransfected with expression vectors for Flag-tagged vIRF-2 (v2), or positive control vIRF-1 (v1)-Flag or vIRF-3 (v3)-Flag, and chitin-binding domain (CBD)-fused USP7 (isoform 2; NM_001286457); empty vectors (vec) were used as negative controls. Cell lysates were subjected to immunoprecipitation (IP) with Flag antibody beads or affinity precipitation (AP) with chitin beads; the respective precipitates were analyzed by immunoblotting with CBD and Flag antibodies to identify coprecipitated USP7-CBD and vIRF-Flag proteins and with the reciprocal antibodies to verify appropriate precipitation of the bait proteins (vIRF-Flag and USP7-CBD). Cell lysates were analyzed analogously to verify appropriate expression of the proteins. (B) USP7 immunoprecipitations from extracts of TRExBCBL1-RTA PEL cells either untreated (latent) or treated with doxycycline (Dox; 1 μg/ml) for 1 day prior to cell harvest. Coprecipitated vIRF-2 was detected using a recently described monoclonal antibody (52). Input endogenous vIRF-2 and USP7 proteins were detected by respective immunoblotting of cell extracts; probing for vIL-6 and β-actin verified lytic induction and provided a loading control, respectively. The observed doublet USP7 bands probably represent different splice isoforms of the deubiquitinase. Ab, antibody; IgG, immunoglobulin G (normal, negative control); U7, USP7. (C) Coprecipitation-based analysis of intracellular USP7 and vIRF-2 association in TRExBCBL1-RTA (BCBL-1) PEL cells. These were infected with a lentiviral vector expressing vIRF-2-Flag, and transduced cells were either left untreated (latent) or treated with doxycycline (1 μg/ml) for 24 h. Cells were treated with DSP cross-linker for 30 min prior to disruption in denaturing buffer (see Materials and Methods), and immunoprecipitations (IP) from diluted extracts were performed using Flag antibody beads directed to Flag-tagged vIRF-2 (v2); bound endogenous USP7 was identified by immunoblotting. Lysates were probed with Flag and USP7 antibodies to verify appropriate protein expression. (Bottom) Doxycycline-induced lytic replication was verified by vIL-6 immunoblotting.

Interactions between USP7 and HHV-8 vIRFs 1, 3, and 4 have been reported (26–28). To test whether vIRF-2 also interacts with USP7, we initially undertook transfection-based coprecipitation experiments employing epitope- and affinity-tagged vIRF-2 (and also vIRFs 1 and 3, positive controls) and USP7 in reciprocal immuno- and affinity precipitations, which detected vIRF-2-USP7 binding (Fig. 1A). To verify interactions of endogenous proteins, a USP7 immunoprecipitation experiment was carried out using extracts of TRExBCBL1-RTA PEL cells, doxycycline inducible for the immediate-early lytic cycle inducer RTA (38); vIRF-2 was identified in the immunoprecipitates from both latently infected and lytically reactivated (doxycycline-treated) cultures, using vIRF-2-specific antibody (39) for immunoblotting (Fig. 1B). Using reciprocal precipitation, Flag-tagged vIRF-2, introduced by lentiviral vector transduction into TRExBCBL1-RTA cells, was able to precipitate USP7 from latently and lytically infected cell extracts (Fig. 1C). In this experiment, cultures were treated with dithiobis-succinimidyl propionate (DSP) cross-linker prior to sodium dodecyl sulfate-mediated cell disruption and protein denaturation, thereby confirming intracellular association of vIRF-2 and USP7 in these naturally infected cells.

**FIG 2:**
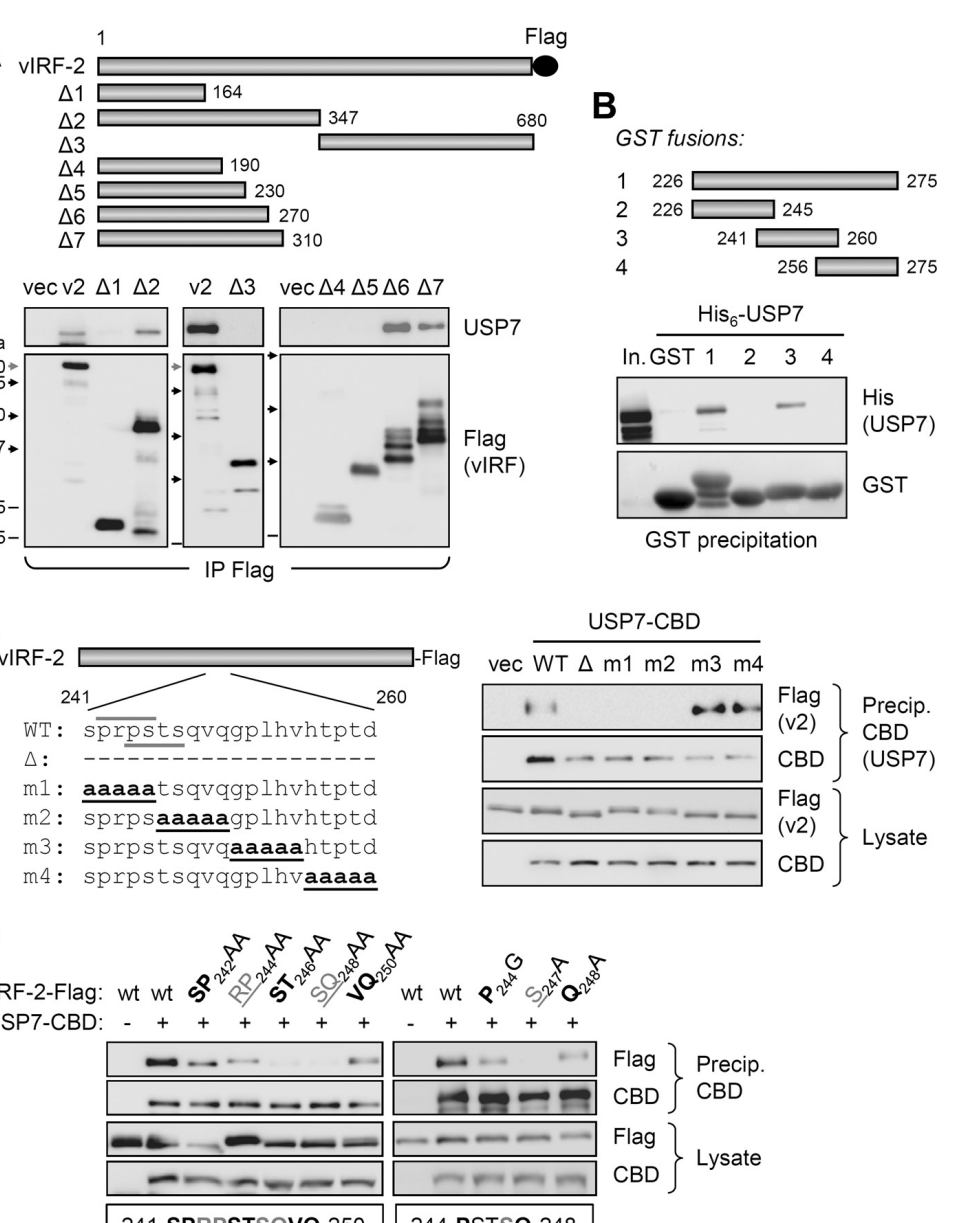
Identification of vIRF-2 region interacting with USP7. (A) Coprecipitation analysis of (endogenous) USP7 interaction with Flag-tagged vIRF-2 and deleted derivatives (Δ1 to Δ7) expressed in transfected HEK293T cells. The migration positions of marker bands on the different blots are indicated by shaded (100 kDa) and black (75, 50, and 37 kDa) arrows and black lines (25 and 15 kDa). Degradation products (multiple bands) are evident for some of the vIRF-2 deletion variants. (B) *In vitro* coprecipitation analysis of interaction between recombinant, bacterially derived and purified His_6_-tagged USP7 and GST-fused vIRF-2 residues 226 to 275 and subfragments 226 to 245, 241 to 260, and 256 to 275. Glutathione bead precipitates were analyzed by USP7 immunoblotting for detection of vIRF-2 fragment-USP7 interactions. In., input His_6_-USP7. (C) Plasmid vectors expressing the indicated vIRF-2 proteins (left) deleted of (Δ) or mutated (m1 to m4) in the 241- to 260-residue USP7-binding region of vIRF-2 were used in transfection-based coprecipitation assays. Coexpressed CBD-tagged USP7 (isoform 2; NP_001273386.1) was precipitated (Precip.) from transfected cell lysates with chitin beads, and precipitates and lysates were analyzed for USP7-interacting and appropriately expressed vIRF-2 (v2) proteins, respectively. CBD immunoblotting confirmed appropriate affinity precipitation and expression of USP7-CBD. (Left) Over/underlined wild-type (WT) sequences correspond to USP7-binding consensus motifs. (D) Similar analysis of double and single point mutations of vIRF-2 residues 241 to 250. The residues targeted for double and single mutations are indicated below the respective sets of immunoblots of precipitates and lysates from the corresponding transfectants.

To identify the region(s) of vIRF-2 involved in USP7 interaction, we generated a series of vectors expressing various Flag-tagged vIRF-2 fragments (Fig. 2A, top), which were tested for their interaction with endogenously expressed USP7 via coprecipitation assay. The resulting data (Fig. 2A, bottom) identified vIRF-2 residues between 231 and 270 as required for interaction with USP7 in the context of successively extended N-terminal sequences, thereby potentially mapping the USP7-interacting region of vIRF-2 to within these 40 residues. Indeed, this region of vIRF-2 and a subfragment containing residues 241 to 260, when fused to glutathione S-transferase (GST) as recombinant proteins, could precipitate bacterially derived and purified His_6_-tagged USP7, demonstrating both sufficiency of these vIRF-2 residues for interaction and direct interaction between the two proteins (Fig. 2B).

We next sought to identify specific residues of vIRF-2 required for the interaction in the context of the full-length protein. Vectors were generated to express vIRF-2 deleted of USP7-binding residues 241 to 260 or containing penta-alanine substitutions across this region (Fig. 2C, left), and these were used in transfection-based coprecipitation assays. The results (Fig. 2C, right) identified residues within amino acid positions 241 to 250 (encompassed by mutations m1 and m2) as required for interaction with USP7. This region contains two overlapping motifs, PRPS and PSTS, matching the previously reported USP7-binding A/PxxS consensus (40–42); the second of the two vIRF-2 motifs was altered by both the m1 and m2 substitutions (Fig. 2C). Using more refined substitution mutagenesis and coprecipitation assays, residues 245 to 247 were identified as important for binding, with S_247_, the terminal residue of the PSTS motif, alone being essential for vIRF-2 interaction with USP7 (Fig. 2D); this is consistent with previously reported analyses of USP7 binding by equivalent motifs (41). It is important to note, however, that P_244_, the first residue of the putative USP7 interaction motif, was not required for binding; mutation of this residue to glycine diminished but did not abolish interaction, similar to the effect of the Q_248_A mutation, outside the consensus USP7 interaction sequence. These results were reproducible. Thus, while the PSTS sequence of vIRF-2 (identical to the USP7-binding motif of MDM2) is involved in interaction of vIRF-2 with USP7, it is unclear whether, in the context of vIRF-2, its binding activity is precisely analogous to similar USP7 interaction motifs identified in other proteins, where the first P (or equivalent A) residue is involved directly in interaction (40–42).

### The N-terminal TRAF-like domain of USP7 is insufficient for vIRF-2 interaction.

**FIG 3:**
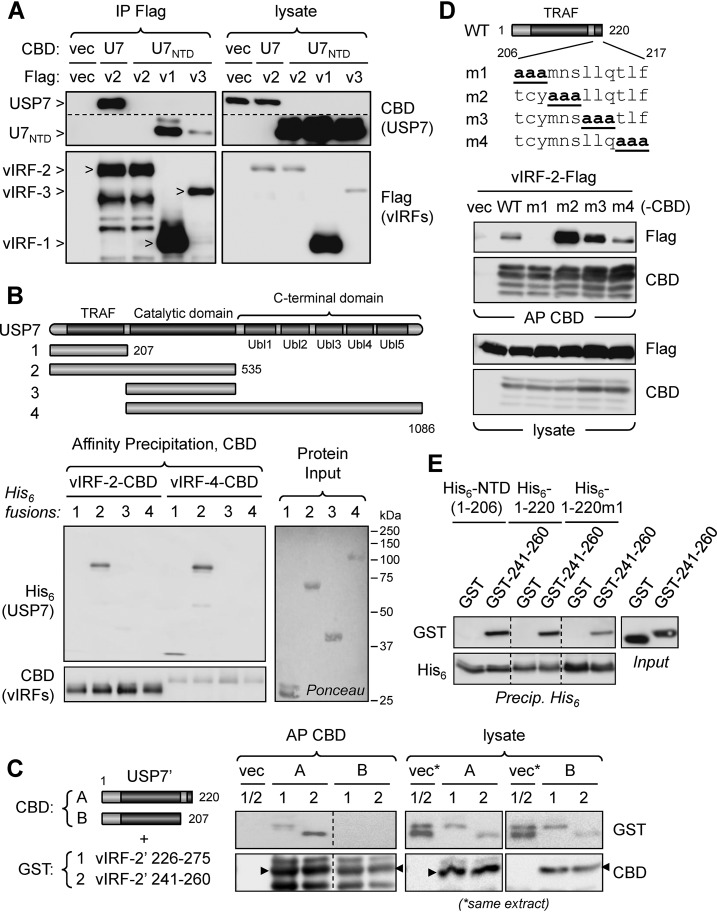
Identification of USP7 region required for interaction with vIRF-2. (A) Transfection-based coprecipitation assays to assess interaction of Flag-tagged vIRF-2 (v2) and positive-control vIRF-1 (v1) and vIRF-3 (v3) proteins, with CBD-fused USP7 NTD (U7_NTD_) and full-length USP7 (U7, positive control; isoform 2; NP_001273386.1). The identities of USP7 bands on the CBD blots and vIRF bands on the Flag blots are indicated (arrowheads). The dotted line indicates a deleted (irrelevant) region of the CBD blot. (B) Coprecipitation analysis of vIRF-2 interactions with individual and combined domains of USP7, derived from bacteria as His_6_-fusion proteins, and CBD fusions of vIRF-2 or vIRF-4 (positive control for USP7 NTD binding) expressed in transfected cell extracts. Chitin bead precipitates were His_6_ immunoblotted for detection of coprecipitated USP7 derivatives. (Right) Input USP7-His_6_ proteins were validated and compared by Ponceau staining. (C) Coprecipitation analysis using eukaryotically expressed, CBD-fused USP7 fragments comprising residues 1 to 220 (A) and 1 to 207 (NTD) (B) (USP7 sequence NP_001273386.1) and bacterially expressed, purified GST fusions of vIRF-2 residues 226 to 275 (1) and 241 to 260 (2) (each containing USP7-binding sequences, as shown in Fig. 2B). The CBD-fused USP7 fragments were affinity precipitated (AP) with chitin beads, and coprecipitated GST-fused vIRF-2 peptides were identified by GST immunoblotting. Empty vector (vec) transfectant extract provided a negative control. Major full-length protein species (appropriately migrating; size markers not shown) are indicated by arrowheads; higher and lower bands likely represent posttranslationally modified and degradation products, respectively. The dotted line in the precipitation blots indicates repositioning of lanes (from the same membrane). (D) CBD-fused residues 1 to 220 of USP7 (WT; isoform 2; NP_001273386.1) or triplet alanine substitutions thereof (m1 to m4) were used in chitin bead affinity precipitations along with Flag-tagged vIRF-2, each protein pair being derived from transfected HEK293T cell lysates. Empty vector (vec) provided a negative control. (E) *In vitro* coprecipitation assay employing bacterially expressed and purified GST-fused vIRF-2 residues 241 to 260, or GST (negative control), and His_6_-tagged USP7 NTD (USP7 residues 1 to 206), USP7 residues 1 to 220, or the m1 variant of the latter (His_6_-1-220m1; see panel D). Dotted lines indicate deleted (irrelevant) lanes. Precip., precipitation.

Having identified the PSTS_247_ motif as involved in vIRF-2 interaction with USP7, we expected that vIRF-2 would interact with the N-terminal domain (NTD) of USP7, as reported for other USP7-binding proteins containing similar interaction motifs (32). This was tested in a coprecipitation assay, employing CBD-fused full-length USP7 or USP7 NTD and Flag-tagged vIRF-2, or vIRF-1 and vIRF-3 positive controls. While the latter vIRFs precipitated the USP7 NTD (U7_NTD_), as expected, vIRF-2-Flag precipitated only full-length USP7 (Fig. 3A). We then generated four His_6_-linked, bacterially expressed, and purified USP7 fragments comprising residues 1 to 207 (N-terminal domain), 1 to 535 (N-terminal plus catalytic domains), 208 to 535 (catalytic domain), and 208 to 1086 (catalytic plus C-terminal domain) and tested their abilities to bind affinity-precipitated vIRF-2-CBD, or vIRF-4-CBD (positive control for NTD interaction), expressed in transfected cell extract. This experiment confirmed the insufficiency of the NTD region of USP7 for vIRF-2 binding (but interaction of NTD with vIRF-4) and the ability of NTD plus catalytic domain sequences to bind vIRF-2 (Fig. 3B). A further binding experiment utilized transfected cell-expressed CBD-tagged USP7 fragments comprising the NTD (residues 1 to 207) or the NTD fused to the first part of the catalytic domain (residues 1 to 220) along with bacterially expressed and purified GST-fused vIRF-2 residues 226 to 275 or 241 to 260 (each containing the USP7-binding region of vIRF-2). This experiment determined that extension of the NTD by 13 residues was sufficient to confer vIRF-2 binding (Fig. 3C). Together, the USP7 mapping data demonstrate that vIRF-2 is distinct from the other HHV-8 vIRFs in its requirement for residues in addition to the USP7 NTD for its interaction with USP7 in these experimental systems.

To identify specific residues of the catalytic domain that were required to confer vIRF-2 binding to the USP7 NTD, triplet alanine scanning mutations were introduced into the region of USP7 between residues 206 and 217 in the context of a CBD-fused USP7 fragment comprising residues 1 to 220 (Fig. 3D, top). Affinity precipitations of the CBD fusions from vIRF-2-Flag-coexpressing transfected cell lysates determined that residues 206 to 208 were required for interaction (Fig. 3D). Interestingly, alanine substitutions of residues 209 to 211 (m2) substantially increased the amount of coprecipitated vIRF-2, suggesting that binding affinity and/or accessibility was enhanced by the triple mutation. In a similar experiment, alanine substitutions of NTD residues known to be important for interactions of USP7 substrates such as p53 and MDM2 (40, 42) led to loss of (W_149_A and DWGFS_152_A_5_) or greatly diminished (R_88_A) interaction of the residue 1 to 220 USP7 fragment with vIRF-2 (data not shown), indicating canonical interaction of the vIRF-2 PSTS motif with the USP7 TRAF-like domain despite the additional requirements for vIRF-2 and its binding region (residues 241 to 260) to interact with USP7.

We also analyzed interaction of vIRF-2 with USP7 using an *in vitro* assay employing bacterially expressed, purified proteins. The assay utilized GST-fused vIRF-2 residues 241 to 260, or GST (negative control), and His_6_-tagged USP7 residues 1 to 206 (NTD), 1 to 220, or the TCY_208_AAA (m1) variant of 1 to 220. All of these fragments, including the m1 variant that was unable to coprecipitate vIRF-2 from transfected cell lysates, were able to interact with the vIRF-2 peptide *in vitro* (Fig. 3E). Possible explanations for the distinct results from the *in vitro* versus *in vivo* assays include binding-enhancing conformational changes exerted by the NTD distal sequences and required intracellularly (or in the presence of cell extract in the binding assay) due to other protein interactions and/or particular USP7 protein conformations effected via eukaryotic cell-specific posttranslational modifications of USP7. Regardless of the underlying mechanism, however, it is clear that the vIRF-2-USP7 interaction is distinct from other reported interactions with USP7 NTD.

### USP7 modulation of TRAF signaling.

**FIG 4:**
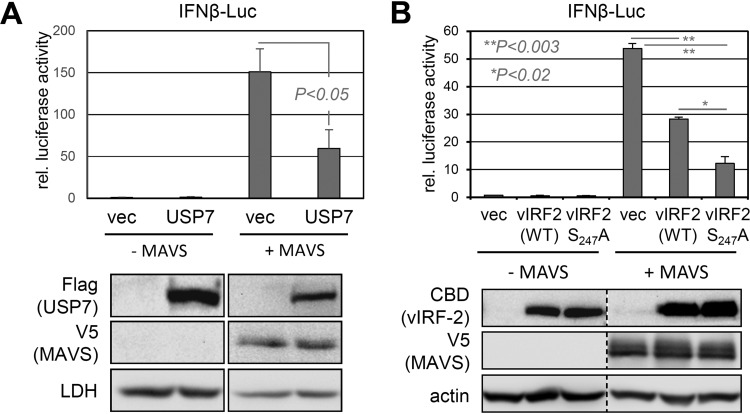
USP7 and vIRF-2 effects on MAVS-mediated signaling. (A) USP7 suppression of MAVS-induced signaling, as measured by IFN-β-luciferase (Luc) reporter assay, was detected in HEK293T cells cotransfected with the reporter and empty vector (vec) or expression plasmid for USP7 (isoform 1; NP_003461.2), with or without addition of MAVS expression vector. Luciferase activity in the extracts was determined by luminescence assay; relative values (means from duplicate transfectants from two experiments; *N* = 4) are shown along with standard deviations of replicate values from the means. Student's *t* test (two tailed) was used to assess statistical significance. Immunoblots below the respective transfectants show equivalent expression of MAVS between the +MAVS samples and appropriate expression of introduced USP7 (samples were derived from one of the transfection replicates). (B) A similar experiment was carried out to assess effects of vIRF-2 or USP7-refractory vIRF-2.S_247_A on MAVS-induced IFN-β promoter induction. The immunoblots below show appropriate expression of the respective proteins from the input plasmids (extracts from duplicate transfectants were pooled). *P* values were obtained by Student's *t* test (two tailed).

USP7-binding TNF receptor-associated factor (TRAF) E3 ubiquitin ligases are essential components of antiviral signaling, They are initiated by a variety of infection-sensing molecular pattern recognition receptors that activate innate immune signaling pathways, leading (in part) to IRF3/7 and NF-κB activation-mediated induction of type I interferons (alpha interferon [IFN-α] and IFN-β) that are critical for host cell defense against infection (43, 44). Another relevant feature of TRAFs in consideration of potential regulation by vIRF-2, via USP7 interaction, is that they are expressed in the cytoplasm, the major location of vIRF-2, as reported by others (23, 45) and confirmed by our own immunofluorescence studies (unpublished data). While *in vitro* interactions of USP7 NTD with TRAFs 1 to 6, intracellular USP7 binding to TRAFs 4 and 6, and USP7 deubiquitination of TRAF6 have been reported (31, 33, 34), biological targeting and deubiquitination of the TRAFs by USP7 have generally not been assessed. In addition to herpes simplex virus ICP0-mediated suppression of antiviral signaling through promotion of USP7 nuclear-cytoplasmic relocalization and consequent deubiquitination of (K63-polyubiquitinated) TRAF6 and associating IKKγ (34), USP7 has been reported to suppress interferon type I induction via K48-polyubiquitin digestion and stabilization of the E3 ubiquitin ligase TRIM27, which targets IRF3/7-activating TBK1 (TRAF family member-associated NF-κB activator binding kinase-1) for K48 polyubiquitination and destabilization via proteasomal degradation (46). We confirmed USP7 suppression of TRAF-mediated innate immune signaling in an assay employing transfected, overexpressed mitochondrial antiviral signaling protein (MAVS), an effector of signaling initiated by retinoic acid-inducible gene I (RIG-I)-like receptors and functioning through TRAFs (44), and a cotransfected IFN-β-luciferase reporter (Fig. 4A). Using a similar approach, we tested the influence of vIRF-2 and its USP7-refractory variant, vIRF-2.S_247_A, on MAVS-activated signaling. Consistent with previous data reporting inhibition of IRF1-, IRF3- and poly(I-C)/MAVS-induced IFN-β-promoter activity by vIRF-2 (23, 47), vIRF-2 was able to inhibit IFN-β promoter-driven luciferase activity (Fig. 4B). Importantly, vIRF-2.S_247_A effected more pronounced inhibition, revealing a positive influence of vIRF-2-USP7 interaction on MAVS-induced signaling. On the basis of these data, we investigated further the regulation of MAVS/TRAF signaling by USP7.

**FIG 5:**
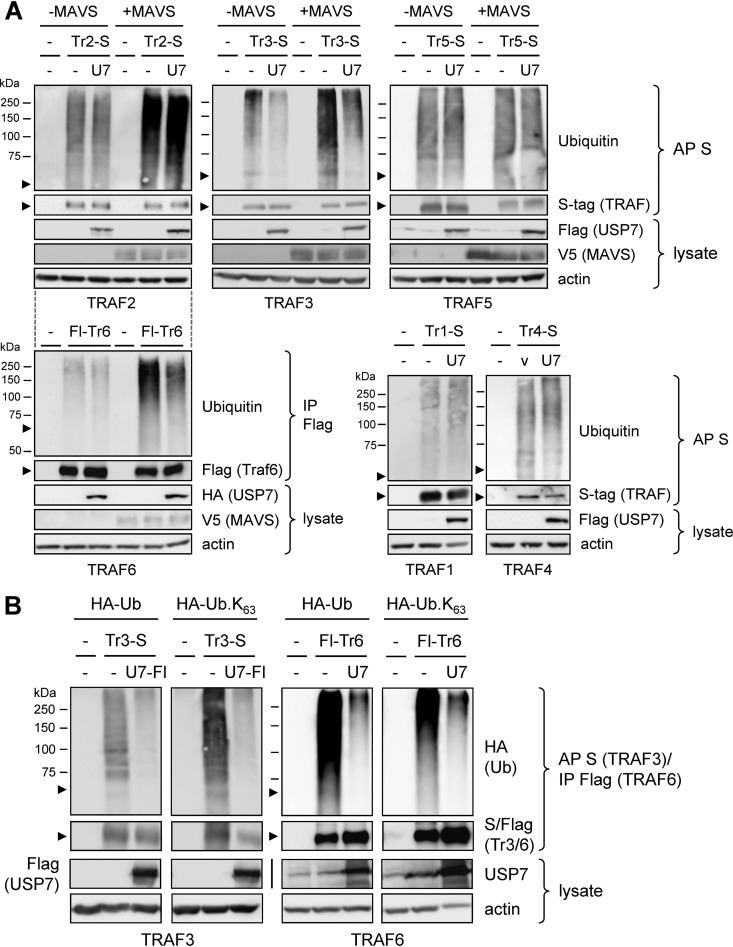
USP7 affects ubiquitination of TRAF3 and TRAF6. (A) HEK293T transfectants expressing S-tagged TRAFs 1 to 5 (Tr1-S to Tr5-S) or Flag-TRAF6 (Flag-Tr6) with or without cotransfected USP7 (U7) and/or MAVS expression vector(s) were analyzed for relative levels of ubiquitination of the respective TRAF proteins. Affinity precipitation (AP; S tag) or immunoprecipitation (IP; Flag) was used to isolate the tagged TRAFs from denatured cell extracts, and the precipitated TRAFs were analyzed by immunoblotting for ubiquitination. Arrowheads indicate the positions of the TRAF proteins on the respective blots. −, empty vector. The dotted lines between the TRAF2 and TRAF6 immunoblot sets indicate the same input plasmids (indicated above the TRAF2 blots) other than the TRAF (and particular USP7 [Flag versus HA tag]) vector used. (B) Similar analyses of TRAF3 and TRAF6 ubiquitination, here in the absence or presence of cotransfected plasmid-expressed HA-tagged wild-type (Ub) or K63-only (Ub.K_63_) ubiquitin, with or without USP7 (isoform 1) expression vector cotransfection. Precipitates were immunoblotted for detection of HA-Ub. Cell lysates were analyzed for appropriate expression of input proteins. U7, USP7; U7-Fl, USP7-Flag.

As TRAF polyubiquitination, induced by MAVS, is required for TRAF activity through signaling complex assembly (44), we assayed by immunoprecipitation and immunoblotting the polyubiquitination of TRAFs 2, 3, 5, and 6 (all activated by MAVS) in appropriately transfected cells expressing each of the TRAFs (S or Flag tagged) in the absence and presence of overexpressed MAVS and with or without overexpressed USP7. The TRAFs were affinity precipitated (S tag) or immunoprecipitated (Flag-TRAF6) from denatured cell extracts and analyzed by immunoblotting for their ubiquitination. Polyubiquitination of TRAFs 3 and 6, specifically, was suppressed in response to USP7 overexpression, thereby identifying TRAF3, in addition to the previously reported TRAF6 (34), as a potential substrate of the deubiquitinase (Fig. 5A). USP7 overexpression correlated with decreased levels of TRAF3 and TRAF6 polyubiquitination in both the absence and presence of MAVS (which, as expected, induced polyubiquitination), thereby confirming the activity of USP7 in the contexts of both unstimulated cells and during activation of MAVS-initiated innate immune signaling. TRAFs 1 and 4, not involved in MAVS signaling and assayed in the absence of its overexpression, were not detectably affected by USP7 overexpression (Fig. 5A, bottom right), indicating that they, along with TRAFs 2 and 5, are not biological targets and/or substrates of the deubiquitinase. A similar experiment was carried out for TRAFs 3 and 6 in cells coexpressing hemagglutinin (HA)-tagged wild-type or K63-only ubiquitin in the absence or presence of overexpressed USP7; TRAFs 3 and 6 were detectably modified by both native and K63-only HA-tagged ubiquitin (detected by HA immunoblotting of TRAF3/6 precipitates) and USP7 effected reductions in each, the latter confirming USP7 regulation of signaling-associated K63-linked polyubiquitination of TRAF3 and TRAF6 (Fig. 5B). The data confirm and extend previously reported USP7-effected deubiquitination of TRAF6 (34) and indicate that TRAF3 also is a target of inactivation by the deubiquitinase.

### Physical interactions of USP7 with TRAF3 and TRAF6 and influence of vIRF-2.

**FIG 6:**
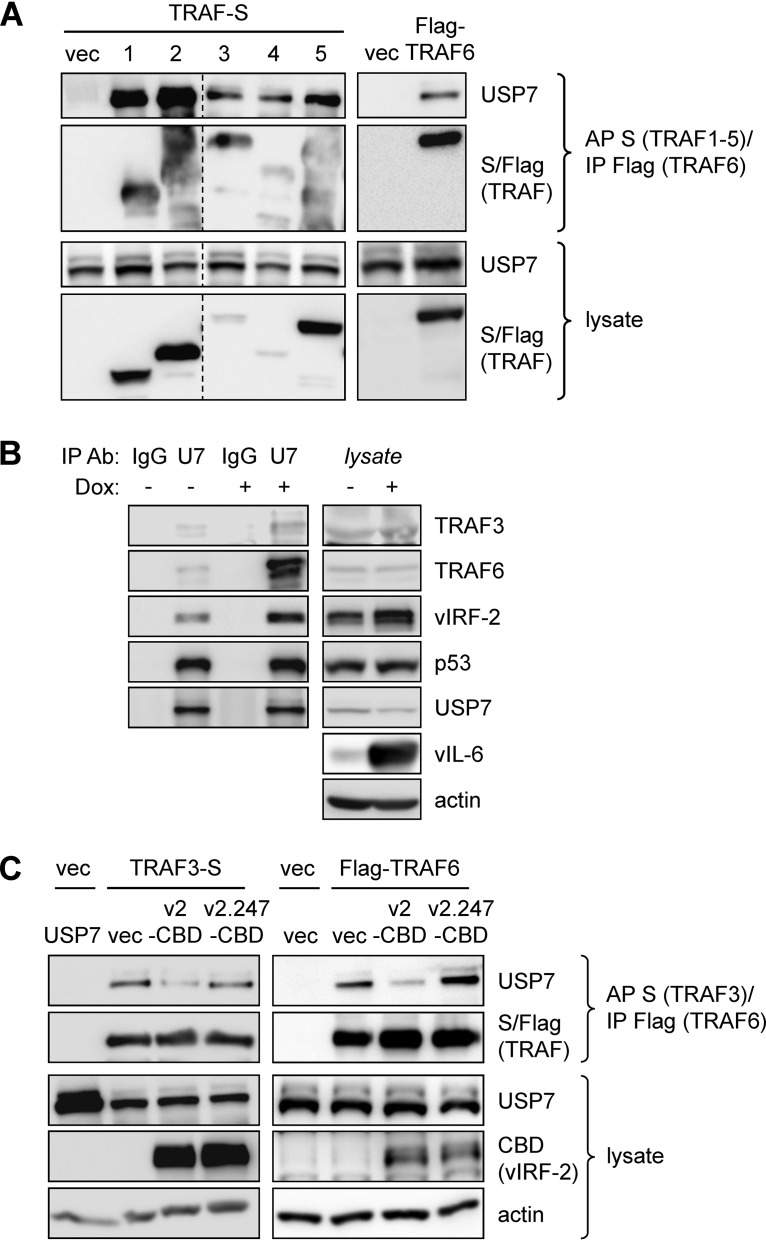
Intracellular interactions of USP7 with TRAF3 and TRAF6 and influence of vIRF-2. (A) TRAF3 and TRAF6, along with TRAFs 1, 2, 4, and 5, were tested by transfection-based coprecipitation assays for their interactions with endogenous USP7 in HEK293T cells. Cells were treated with DSP cross-linker for 30 min prior to cell disruption. Affinity precipitation (AP) of S-peptide (TRAFs 1 to 5) or immunoprecipitation (IP) of Flag (TRAF6) from denatured cell extracts was followed by immunoblotting for USP7. Cell lysates were analyzed by immunoblotting for input TRAFs and endogenous USP7 (loading control). vec, empty vector; dotted line indicates repositioning/reordering of lanes. (B) A reciprocal coprecipitation experiment (again involving DSP cross-linking prior to cell disruption and protein denaturation) was carried out by immunoprecipitating USP7 from extracts of PEL (TRExBCBL1-RTA) cells, either latently infected (untreated) or induced into the lytic cycle with doxycycline, and immunoblotting to detect cosedimented TRAF3 and TRAF6 (and also vIRF-2). Immunoblotting for p53 provided a positive control for interaction with USP7. Lysates were probed to determine levels of TRAF3, TRAF6, USP7, p53, and vIRF-2 in latent versus lytic cells; immunoblotting of cell lysates for detection of vIL-6 verified lytic induction in the presence of doxycycline, applied for 36 h before cell harvest. (C) A coprecipitation experiment similar to that of panel A was carried out using either TRAF3-S or Flag-TRAF6 in the absence (empty vector, vec) or presence of transfected vector-expressed vIRF-2-CBD (v2-CBD) or vIRF-2.S_247_A-CBD (v2.247-CBD).

While the above-described data indicated that TRAF3, in addition to TRAF6, is a substrate of USP7, we wanted to test the interaction between the two proteins; previously, interaction between TRAF3 and the N-terminal TRAF-like domain of USP7 was detected *in vitro* (31). In transfection-based coprecipitation assays, we found that epitope-tagged TRAF3 and TRAF6, in addition to TRAFs 1, 2, 4, and 5, were able to precipitate endogenous USP7 from HEK293T cell lysates (Fig. 6A). Furthermore, endogenous TRAFs 3 and 6, in addition to vIRF-2 (and p53, a positive control), were immunoprecipitated with USP7 from PEL (TRExBCBL1-RTA) cell lysates (Fig. 6B). For both experiments, proteins were cross-linked prior to cell disruption and protein denaturation, enabling the verification of bona fide interactions in the intact cells. Notably, the USP7-TRAF3/6 interactions in PEL cells were enhanced in lytically reactivated relative to latently infected cultures, indicating enhanced suppression by USP7 of TRAF3 and TRAF6 polyubiquitination and signaling in the context of productive replication (and activated TRAFs). In transfected HEK293T cells, vIRF-2 led to decreased interaction of USP7 with TRAF3 and TRAF6, while USP7 interaction-defective vIRF-2.S_247_A had no effect (Fig. 6C). These data are indicative of competitive interactions of vIRF-2 and the potential for vIRF-2 to influence TRAF3/6-mediated signal transduction via suppression of USP7-TRAF3/6 binding and associated TRAF deubiquitination.

### Regulation of TRAF ubiquitination by vIRF-2.

**FIG 7:**
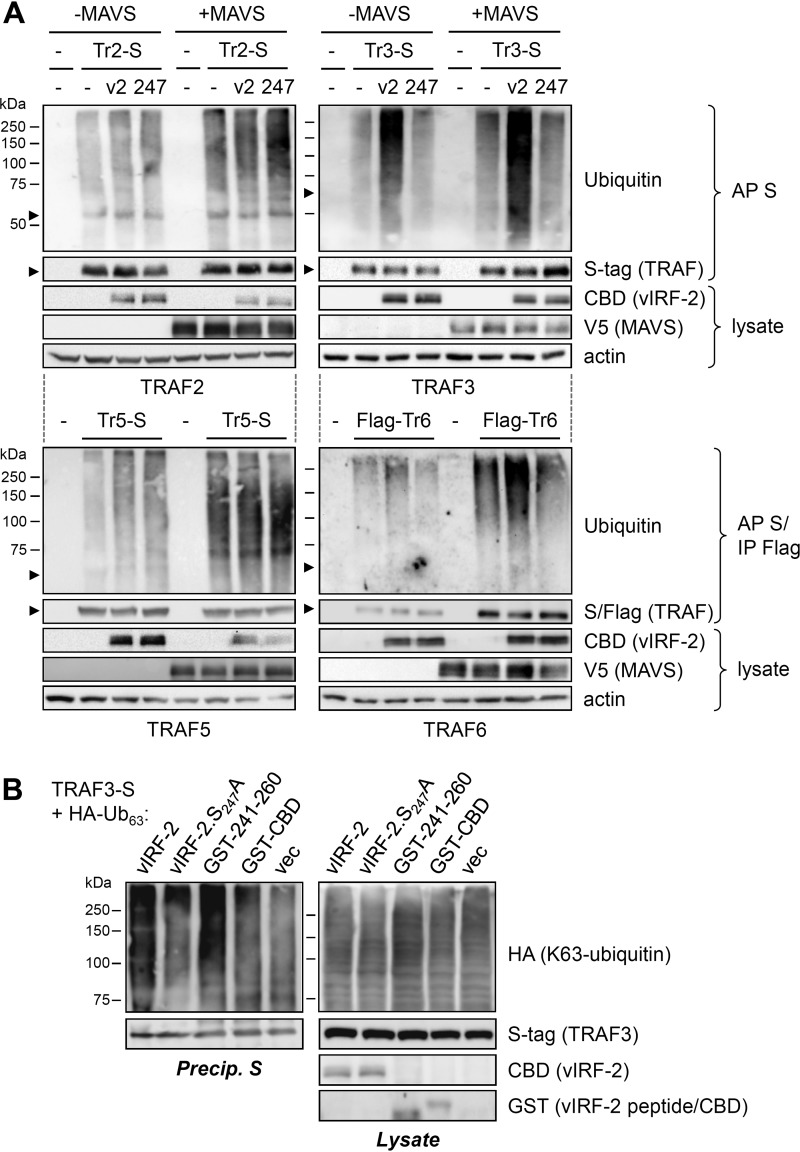
USP7 binding-dependent regulation of TRAF signaling by vIRF-2. (A) Effects of vIRF-2 on regulation of resting and MAVS-activated TRAF ubiquitination was tested in HEK293T-based transfection assays. Expression vectors for TRAFs 2, 3, 5, and 6 were cotransfected with either empty vector (−) or with vIRF-2 (v2) or vIRF-2.S_247_A (247) expression plasmid, with or without MAVS expression vector. TRAFs were affinity precipitated (AP) with S-protein beads (S-tagged TRAF2 [Tr2-S], TRAF3 [Tr3-S], and TRAF5 [Tr5-S]) or immunoprecipitated (IP) with Flag antibody beads (Flag-TRAF6 [Flag-Tr6]) from denatured cell lysates, and precipitates were immunoblotted with ubiquitin antibody. Lysates were checked for protein expression from the input vectors; β-actin immunoblotting provided a loading control. Arrowheads indicate the migration positions of the respective TRAF proteins. (B) A similar experiment was undertaken to analyze K63-linked polyubiquitination of TRAF3, effected by overexpression of HA-tagged K63-only ubiquitin (HA-Ub_63_), in response to expression of CBD-tagged vIRF-2 or vIRF-2.S_247_A, GST-fused USP7-binding fragment (241-260) of vIRF-2, or GST-CBD or empty vector (vec) negative controls. S-protein bead precipitates from denatured transfected cell lysates were analyzed by HA immunoblotting for K63 polyubiquitination of TRAF3-S; lysates were checked for expression of input proteins (GST-CBD band not shown).

In view of apparent vIRF-2 competition for USP7 interactions with TRAF3 and TRAF6, the detection of USP7 modulation of polyubiquitination of TRAFs 3 and 6 (Fig. 5), and evident USP7 binding-dependent activation of MAVS-stimulated signaling by vIRF-2 (Fig. 4B), we assessed the effect of vIRF-2 and its targeting of USP7 on TRAF polyubiquitination. Transfection- and coprecipitation-based assays were carried out, as before, to detect polyubiquitination of TRAFs 2, 3, 5, and 6 in the absence and presence vIRF-2 or vIRF-2.S_247_A, with or without MAVS overexpression. The results revealed augmentation of TRAF3 and TRAF6 polyubiquitination in the presence of vIRF-2, but not vIRF-2.S_247_A (unable to bind USP7 and compete for USP7-TRAF3/6 interactions), and the selective effect of vIRF-2 on these two MAVS-activated TRAFs (Fig. 7A). A similar analysis of TRAF3 ubiquitination in the presence of overexpressed HA-tagged K63-only ubiquitin demonstrated the ability of vIRF-2 and USP7-binding residues 240 to 261 (fused to GST), but not vIRF-2.S_247_A and GST-CBD (negative control), to augment signaling-relevant K63-linked polyubiquitination of TRAF3 (Fig. 7B). Together with the binding competition data (Fig. 6C), these TRAF ubiquitination analyses provide evidence of vIRF-2 positive and selective regulation of TRAF3- and TRAF6-mediated signal transduction through inhibition of USP7-TRAF3/6 interactions and consequent TRAF3/6 deubiquitination.

### Functions of vIRF-2 and vIRF-2-USP7 interaction in HHV-8 biology.

**FIG 8:**
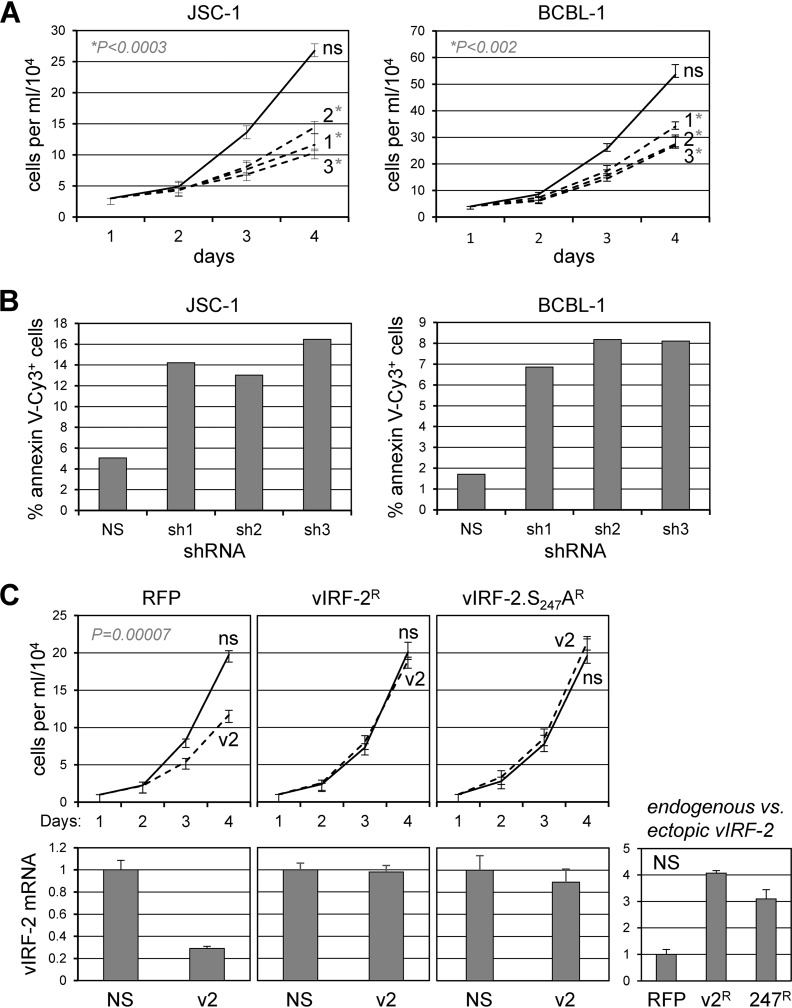
Contributions of vIRF-2 and vIRF-2-USP7 interaction to latently infected PEL cell growth and viability. (A) Growth curves (viable cell density/time) for JSC-1 and BCBL-1 (TRExBCBL1-RTA) cells transduced with lentiviral vectors expressing vIRF-2 mRNA-targeting shRNAs (1 to 3) or nonsilencing shRNA control (ns). Data were derived from triplicate cultures for each condition; error bars show standard deviations from the means. Statistical significance between values for each vIRF-2 shRNA and control shRNA samples was determined by Student's *t* test (two tailed); *P* values for vIRF-2 shRNA 1-, 2-, and 3-reduced cell densities (relative to NS shRNA-transduced controls) at day 4 were 0.00021, 0.00013, and 0.00002 for JSC-1 cultures and 0.00127, 0.00065, and 0.00108 for BCBL-1 cultures. (B) Measurements of apoptosis by Cy3-annexin V staining of day 4 cultures (pooled for each shRNA) shown in panel A. Cells were counterstained with Hoechst dye to identify nuclei, and the percentages of Cy3-annexin V^+^ cells were calculated from data derived from multiple random fields per sample (>900 total cells/sample). (C) vIRF-2 mRNA-specific shRNA3 (v2) or control (ns) shRNA was expressed via lentiviral vector transduction in BCBL-1 (TRExBCBL1-RTA) cells pretransduced with lentiviruses expressing shRNA3-resistant mRNAs encoding vIRF-2 (vIRF-2^R^) or vIRF-2.S_247_A (vIRF-2.S_247_A^R^) or with RFP-expressing lentiviral vector (RFP, control). Cell growth was monitored as outlined for panel A. Charts below the growth curves show the relative levels of vIRF-2 transcripts in the respective PEL cultures, as determined by RT-qPCR applied to RNA extracted from terminal (day 4) cultures; the rightmost chart shows the levels of vIRF-2 mRNA in vIRF-2 and vIRF-2.S_247_A vector-transduced cells (absent depletion) relative to the level in the RFP-expressing control vector-transduced cells. For all charts, average values from triplicate samples are shown, with error bars indicating standard deviations of the individual from the average values. The *P* value for the growth effect of uncomplemented (RFP) vIRF-2 depletion was obtained by Student's *t* test (two tailed).

It has been reported very recently (during conclusion of our own studies here) that vIRF-2 inhibits lytic gene expression during lytic reactivation in and *de novo* infection of endothelial cells and inhibits virus production in the former (39); these are the only published phenotypic studies of vIRF-2. It is notable that in the context of PEL cells, naturally infected with HHV-8, vIRF-2 transcripts and protein have been detected in both latency and lytic infection (24, 39, 48). We first tested for potential effects of vIRF-2 depletion on the growth of latently infected PEL cells. Lentiviral vectors expressing three short hairpin RNAs (shRNAs) were made and used, along with nonsilencing (NS) shRNA-expressing lentivirus (control), to transduce JSC-1 and BCBL-1 (TRExBCBL1-RTA) cultures. Despite modest reductions of vIRF-2 mRNA (41 to 64% in the JSC-1 and 49 to 66% in the BCBL-1 cultures; data not shown), each of the vIRF-2-directed shRNAs effected substantial depletion of vIRF-2 protein levels (13% to 23% and 16% to 27% in JSC-1 and BCBL-1 cells, respectively; data not shown) and a significant reduction, relative to the control, of viable cell densities over the 3-day experiment (Fig. 8A). Cells collected on the last day were analyzed by annexin V-Cy3 and Hoechst staining to determine rates of apoptosis in vIRF-2-depleted versus control cultures; these data revealed a significant increase in apoptosis in the former (Fig. 8B). To assess the specific relevance to the detected vIRF-2 latent phenotype of USP7 targeting by vIRF-2, we compared the abilities of vIRF-2 and USP7-refractory vIRF-2.S_247_A, each encoded by lentiviral vector-expressed shRNA-resistant mRNAs, to functionally complement vIRF-2 depletion. Monitoring of viable cell densities over 3 days revealed that both wild-type vIRF-2 and vIRF-2.S_247_A were able to complement depletion of endogenous vIRF-2 (growth inhibitory in the context of uncomplemented cells, transduced with red fluorescent protein [RFP]-encoding lentivirus) (Fig. 8C), indicating that vIRF-2-USP7 interaction does not contribute significantly to the proviability functions of vIRF-2 in these latently infected cells. A caveat, however, is that the levels of vIRF-2 mRNA in RFP versus wild-type or S_247_A vIRF-2-transduced cells were substantially higher (3- to 4-fold) in the latter (Fig. 8C, bottom-right chart), raising the possibility that any contributions of vIRF-2-USP7 interaction to cell viability could conceivably be masked by increased (and threshold-exceeding) compensating activities of vIRF-2.

**FIG 9:**
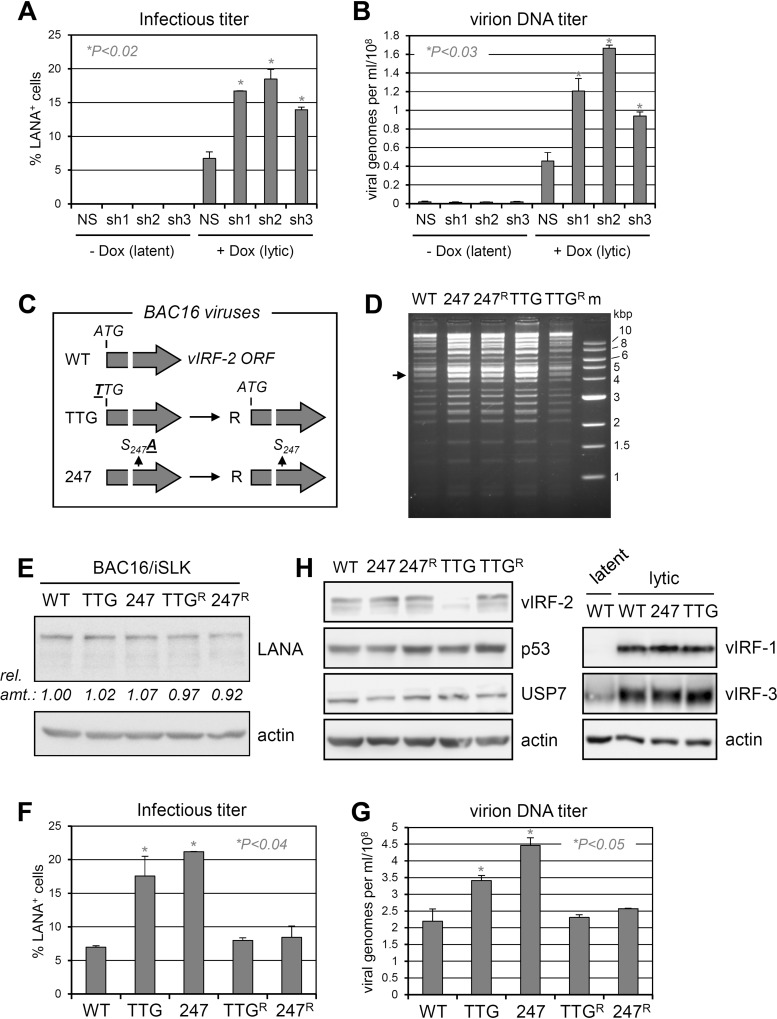
Contributions of vIRF-2 and its interaction with USP7 to productive replication. (A) Infectious titers of HHV-8 produced from lytically reactivated (doxycycline-treated) TRExBCBL1-RTA PEL cells in the absence and presence of vIRF-2 depletion. Duplicate cultures were transduced with lentiviral vector-delivered nonsilencing (NS) control shRNA or vIRF-2 mRNA-targeting shRNAs (sh1, sh2, or sh3), and the media from doxycycline-treated or untreated cultures were harvested 4 days after lytic induction with doxycycline (1 μg/ml for first 24 h) or mock treatment. These inocula were used to infect naive iSLK cells, and the percentages of infected cells were determined by LANA immunostaining (see Materials and Methods). *P* values (Student's *t* test, two tailed) for effects of shRNAs 1, 2, and 3 in lytically reactivated cells are 0.005, 0.011, and 0.010, respectively. (B) Samples of the same media were used to determine the yields of encapsidated (DNase I-resistant) HHV-8 genomic DNA by qPCR using primers directed to LANA open reading frame (ORF73) sequences (see Materials and Methods). *P* values (Student's *t* test, two tailed) for effects of shRNAs 1, 2, and 3 are 0.023, 0.003, and 0.021, respectively. (C) Diagrammatic representation of mutations introduced into BAC16 to generate the respective vIRF-2 knockout (TTG), vIRF-2.S_247_A-substituted (247), and revertant (R) viruses used in subsequent phenotypic analyses. (D) Restriction analyses (NheI + XhoI) of wild-type (WT), point-mutated (initiator codon ATG to TTG [TTG] and vIRF-2 S_247_-to-A_247_ codon mutation [247]), and wild-type-reverted (TTG^R^ and 247^R^) BAC16 HHV-8 genomes, showing overall integrity of the genomes (indistinguishable from wild-type BAC16). The arrow indicates the position of the 4.3-kbp fragment containing the vIRF-2 gene. (E) LANA immunoblotting of extracts of iSLK cultures infected with the same infectious doses (determined by LANA staining, as outlined for panel A) of the different viruses and subsequently hygromycin selected to remove uninfected cells. These cultures were used, in duplicate, for lytic induction and assessments of infectious virus titers (F) and encapsidated viral DNA yields (G) following 9 days of doxycycline and sodium butyrate treatment (see Materials and Methods). *P* values (determined by two-tailed Student's *t* test) for TTG and 247 effects, relative to the WT, on lytic replication are 0.037 and 0.0001 for data in panel F and 0.049 and 0.018 for data in panel G. (H) Immunoblots (left) of extracts from lytically reactivated BAC16-infected iSLK cultures (harvested 3 days postinduction), showing lack of detectable full-length vIRF-2 expression (but possibly some low-level expression of truncated, alternatively initiated vIRF-2) by the TTG mutant and equivalent expression of vIRF-2 and vIRF-2.S_247_A by the respective wild-type/revertant and 247 viruses. USP7 and β-actin immunoblotting provided loading controls; p53 was probed to identify any modulation by vIRF-2 or its interaction with USP7. The blots on the right show (expected) vIRF-2 specificity of the TTG and S_247_A mutations, with unaffected expression of vIRF-1 and vIRF-3 in the respective extracts.

Lytic functions of vIRF-2 were tested first in lytically reactivated (doxycycline-induced) TRExBCBL1-RTA cells (38). Depletion of vIRF-2 led to a substantial increase in viral titers, measured both by infectious titration assay involving latency-associated nuclear antigen (LANA) staining of PEL media-inoculated iSLK cells (Fig. 9A) and by quantitation of encapsidated viral genomes by quantitative PCR (qPCR) of DNase I-resistant HHV-8 genomic DNA (Fig. 9B). These data demonstrated suppression of lytic replication by vIRF-2, reflective of the effect on lytic replication of vIRF-3 (27), which is, like vIRF-2, expressed in latently infected PEL cells (24, 39, 48). To investigate the generality of the vIRF-2 lytic phenotype and to facilitate biological analysis of vIRF-2-USP7 interaction specifically, bacmid (BAC16)-based recombinant viruses were generated containing either mutation of the initiator ATG codon (ATG to TTG) or of codon 247 to specify USP7-refractory vIRF-2.S_247_A (Fig. 9C); these changes were reverted to wild-type sequences to generate control repaired (R) viruses to ensure that no other phenotypically significant changes occurred during the bacterially based genetic manipulations of the BAC16 genome (39, 49) (see Materials and Methods). The gross integrities of each viral genome was checked by restriction analysis (Fig. 9D), and the introduced genetic changes were verified by sequencing. Viruses reconstituted from electroporated iSLK cells (doxycycline inducible for viral lytic transcriptional regulator RTA [50]) were then used to infect, at equal infectious doses, naive iSLK cells to generate normalized cultures, expressing equivalent levels of latency-associated nuclear antigen per cell (Fig. 9E) (after drug selection for HHV-8^+^ cells; see Materials and Methods). Lytic reactivation was induced by treatment of the cultures with doxycycline and sodium butyrate, and culture media were collected (and replenished) over the course of 9 days to evaluate cumulative, total virus yields. The media were analyzed for relative infectious titers and encapsidated viral genome yields, with these assays producing similar results. Ablating vIRF-2 increased virus yields, consistent with the depletion-based studies in PEL cells and the recent endothelial cell-derived findings of Koch et al. (39), and the same phenotype was effected by abrogation of USP7 interaction by the S_247_A mutation (Fig. 9F and G). Immunoblotting of lysates from similarly infected and induced iSLK cultures harvested at 3 days postinfection confirmed that the ATG-to-TTG mutation led to effective loss of vIRF-2 expression rather than to appreciable levels of truncated translation products reported to be initiated from downstream ATG codons (39) and also verified equivalent levels of expression of vIRF-2 and vIRF-2.S_247_A (Fig. 9H, left). Interestingly, levels of p53 were not detectably altered as a function of vIRF-2-USP7 interaction (WT versus vIRF-2.S_247_A), indicating that activities of vIRF-2 via USP7 are mediated independently of p53 regulation. Additional immunoblotting for vIRF-1 and vIRF-3 confirmed that expression of these replication-regulatory vIRFs were unaffected by the vIRF-2 TTG and S_247_A mutations (Fig. 9H, right). The phenotypic data from these genetic analyses confirm the replication-inhibitory activity of vIRF-2 and the contribution, in whole or part, of USP7 targeting to this function.

### vIRF-2-regulated TRAF activation and interferon induction in the context of infection.

**FIG 10:**
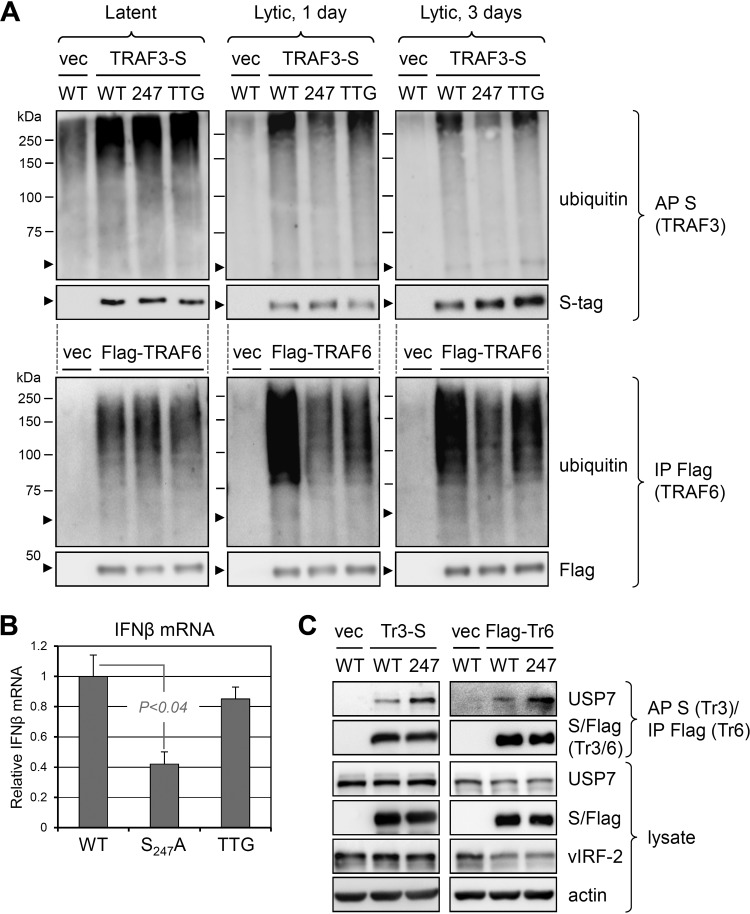
Influence of vIRF-2 and vIRF-2-USP7 interaction on innate immune signaling in the context of lytic infection. (A) Precipitation-immunoblot analyses of (lentivirally transduced, tagged) TRAF3 and TRAF6 ubiquitination during lytic reactivation in doxycycline- and sodium butyrate-treated iSLK cells infected with wild-type (WT), vIRF-2.S_247_A-expressing (247), or vIRF-2-null (TTG) BAC16 virus. Replicate cultures were harvested at day 0 (uninduced, latent) or at 1 or 3 days after lytic induction. Experimental procedures for precipitation were as outlined in the legends to Fig. 5 and 7. (B) Relative levels of expression of interferon-β (IFN-β) transcripts, as determined by RT-qPCR, in lytically reactivated iSLK cells infected with wild-type (WT), vIRF-2.S_247_A-expressing (S_247_A), or vIRF-2 initiator ATG-mutated (TTG) BAC16 virus. Data were derived from duplicate cultures; the average level of IFN-β mRNA in BAC16.vIRF-2.S_247_A- and BAC16.vIRF-2.TTG-infected cells harvested 2 days after lytic induction are expressed relative to levels (set at 1) in parallel wild-type virus-infected cells (error bars indicate standard deviations from the mean values; statistical significance was determined by two-tailed Student's *t* test). (C) Interactions of TRAF3 and TRAF6 with USP7 in iSLK cells infected with BAC16 (WT) or BAC16.vIRF-2.S_247_A (247). The HHV-8^+^ iSLK cultures were transduced with TRAF3-S- or Flag-TRAF6-encoding lentiviral vector, allowing S-protein affinity precipitation (AP) or Flag-immunoprecipitation (IP) from cell lysates 2 days after lytic induction; precipitates were probed for detection of TRAF-interacting USP7 and precipitated TRAFs 3 and 6 (Tr3 and Tr6), and equivalent expression of wild-type and S_247_A vIRF-2 proteins was verified by immunoblotting of cell lysates.

Our transfection-based data indicated the regulation of TRAF3- and TRAF6-mediated signal transduction by vIRF-2 interaction with USP7. To determine the influence of vIRF-2 targeting of USP7 on TRAF3 and TRAF6 polyubiquitination in the context of infection, we compared the ubiquitination status of these TRAFs, expressed in tagged form from lentiviral vectors, in lytically reactivated iSLK cells infected with wild-type or vIRF-2.S_247_A-substituted viruses. The data obtained from this analysis (Fig. 10A) reflected the results of the corresponding transfection experiments (Fig. 7A), in that the S_247_A mutation led to decreased polyubiquitination of TRAFs 3 and 6, indicative of suppression of their activities. Consistent with these data, reverse transcription-qPCR (RT-qPCR)-determined levels of IFN-β mRNA were substantially reduced in the BAC16.vIRF-2.S_247_A-infected cells relative to those infected with wild-type virus (Fig. 10B). Curiously, TRAF3/6 ubiquitination and IFN-β mRNA levels in lytically reactivated iSLK cells infected with vIRF-2-knockout virus (BAC16.vIRF-2.TTG) were not substantially affected (TRAF3 and IFN-β) or were intermediate (TRAF6) between those detected in cells infected with wild-type and BAC16.vIRF-2.S_247_A viruses (Fig. 10A and B). These data suggest that in addition to the positive effects of vIRF-2-USP7 interaction on TRAF activation and signaling, there is at least one counteractivity of vIRF-2 on TRAF3/6 ubiquitination and downstream regulation of IFN-β induction. However, it is conceivable that the small amount of truncated (likely alternatively initiated) vIRF-2 protein detected in BAC16.vIRF-2.TTG-infected cells (Fig. 9H) is functionally sufficient for the observed effects, notwithstanding the clear replication phenotype associated with the TTG mutant.

Finally, we compared USP7-TRAF3/6 interactions in the context of iSLK cells infected lytically with wild-type or vIRF-2.S_247_A BAC16 viruses. TRAFs 3 and 6 were introduced as S-peptide- or Flag-tagged proteins, and associated, coprecipitated USP7 was identified by immunoblotting. USP7-TRAF binding was more pronounced in cells infected lytically with the vIRF-2.S_247_A-expressing virus than in those infected with wild-type virus (Fig. 10C), consistent with the previous transfection-based data (Fig. 6C) showing USP7-TRAF3/6 binding competition by vIRF-2; no vIRF-2.S_247_A-associated change in USP7-TRAF3/6 interaction was detected in latently infected cells, lacking vIRF-2 expression (data not shown). Our data indicate that in the context of infection also, vIRF-2 competes for USP7 interactions with TRAFs 3 and 6 and consequently promotes their polyubiquitination and associated signal transduction.

## DISCUSSION

The present finding of USP7 targeting by vIRF-2, in addition to interactions of HHV-8 vIRFs 1, 3, and 4 with the deubiquitinase, is intriguing, especially as the biological consequences of these interactions appear to be somewhat contradictory and counterintuitive. For example, the present finding of stimulation of TRAF3- and TRAF6-mediated antiviral signaling by vIRF-2 seems to be in functional opposition to the downstream inhibitory effects of vIRF-2 on this pathway, in part via inactivation of phospho-IRF3 by promotion of its cleavage by caspase 3 (23) and also the inhibitory effects of vIRF-1 and vIRF-3 on such signaling through suppression of IRF-directed transcription initiation complex formation (18–21, 51). With respect to vIRF functions through USP7 binding, specifically, the apparently opposing functions of the vIRFs are further evident, with vIRF-1 targeting of USP7 promoting HHV-8 productive replication (in the context of PEL and iSLK cells) and vIRF-2 and vIRF-3 interactions with the deubiquitinase effecting decreased infectious virus and nucleated virion yields (27 and this study). With regard to latency activities in PEL cells, our studies here have failed to identify USP7 interaction-specific effects of vIRF-2 despite proviability activity of vIRF-2 itself, contrasting with proviability functions of vIRF-1 and vIRF-3 through their interactions with USP7 (27). These distinguishable functions of the respective vIRF-USP7 interactions demonstrate clearly the distinct activities and underlying mechanisms associated with USP7 targeting by the different vIRFs. At present, one can only speculate about the possible advantages to the virus of activation of TRAF3- and TRAF6-mediated signaling, which would generally be expected to be detrimental to virus replication. It is possible that activation of such signaling promotes virus latency, as has been proposed by Koch et al. (39), notwithstanding the demonstrable negative impact on virus production upon lytic reactivation; overall, perhaps such negative consequences are balanced by the need to regulate appropriately latent versus lytic infection. Another possibility is that in the context of the host, restriction of viral burst and lytic antigen expression could help to modulate the magnitude of the immune response to productive replication, with overall benefit for virus primary infection and/or virus persistence. It is also possible that counterregulation by vIRF-2 and other vIRFs of the innate immune response to virus infection allows the fine-tuning of signaling pathways to enable appropriate regulation of latent versus lytic replication and to establish conditions conducive to each, depending on particular intracellular and microenvironmental contexts. The latter point would provide an explanation for the multiple, opposing effects by vIRF-2 on innate immune responses: (i) positive regulation of TRAF3/6 signaling via vIRF-2-USP7 interaction (evident by comparisons of TRAF3/6 ubiquitination in wild-type and 247 virus-infected cells; Fig. 10A); (ii) potential countering negative regulation of TRAF3/6 signaling by vIRF-2 (suggested by the relative effects of the 247 and TTG mutations on TRAF3/6 ubiquitination; Fig. 10A) through an unidentified mechanism; (iii) suppression of related innate immune signaling through phospho-IRF3 cleavage and other mechanisms (23); and (iv) induction of interferon-stimulated genes, notably IFITs 1, 2, and 3, by vIRF-2, apparently independently of IFN-I induction (39). Further investigations are required and warranted to determine the effects of these different activities of vIRF-2 on cellular and HHV-8 biology.

It is commonly assumed, in part on the basis of transfection and/or overexpression studies, that viral protein targeting of USP7 will negatively impact p53 expression, generally induced upon virus infection and replication and inhibitory to virus production. However, our own studies in the context of infected cells have failed to identify any USP7 binding-dependent effects of vIRF-1 and vIRF-3 on p53 levels in latently infected PEL cells and during lytic reactivation in these and/or HHV-8^+^ iSLK cells; indeed, depletion of USP7, which regulates p53 stability through the relative deubiquitination of p53 and its E3 ubiquitin ligase MDM2 (29, 52), while phenotypically significant in PEL latency and HHV-8 productive replication, did not detectably alter p53 expression (27). Also in the present study, p53 levels were indistinguishable between iSLK cultures infected with wild-type HHV-8 and mutated virus expressing USP7-refractory vIRF-2.S_247_A (Fig. 9H), suggesting that regulation of p53 is not effected via vIRF-2 interaction with the deubiquitinase. On the other hand, we have identified physical and functional interactions of USP7, namely, those with TRAF3 and TRAF6, that are affected by vIRF-2 in the contexts of transfected and lytically infected cells. The functional consequences of vIRF-2-USP7 interaction with respect to activating (K63-linked) polyubiquitination of these TRAFs and downstream type I interferon gene expression were evident in transfected cells in which vIRF-2 was expressed in isolation and in infected cells in which vIRF-2 and other viral lytic proteins were expressed naturally from viral genomes. Thus, our data identify likely effectors of vIRF-2 activity via USP7 interaction; to our knowledge, this represents the first example of vIRF disruption of USP7-protein interaction and resulting functional and phenotypic consequences. Although this finding advances understanding of the mechanisms of vIRF activity via USP7 targeting, it is evident that there are many other USP7-cellular and -viral protein interactions that potentially can be (and probably are) regulated via vIRF-USP7 binding. It is important to appreciate that effects of the vIRFs through their interactions with USP7 may promote as well as disrupt interactions of USP7 with its substrates, analogous to the situation reported for the stabilization of the HHV-8 ORF33-encoded tegument protein (pORF33) by promotion of pORF33-USP7 interaction by USP7-binding pORF45 (37). Thus, while our present data identify one mechanism of USP7-associated vIRF-2 activity, i.e., via TRAFs 3 and 6, there are likely many other means by which vIRF-2, and the other vIRFs, regulate cellular activities impacting virus biology through USP7 binding.

Our current reporting of negative effects of vIRF-2 on HHV-8 productive replication is consistent with recently published work on vIRF-2 activity in HHV-8-infected endothelial cells (39). This study found that viral early gene expression and virus yields from lytically reactivated huARLT cells were promoted by vIRF-2 gene deletion. Productive replication was enhanced approximately 10-fold, appreciably beyond the 2- to 3-fold increases that we observed in our PEL and iSLK cell-based experiments. Therefore, vIRF-2 activities, in sum, appear to limit HHV-8 productive replication, at least in culture. This could reflect a natural function of vIRF-2 in promoting latency via limiting early lytic gene expression, as posited by Koch et al. (39). This also may be the case for vIRF-3, which is expressed as a bona fide latency gene in PEL cells, at least, and is clearly inhibitory to productive replication (12, 27, 48). However, whether this can explain fully the replication-inhibitory effects of these vIRFs in the contexts of TRExBCBL1-RTA and iSLK cells, in which the immediate-early transactivator RTA is expressed in response to doxycycline treatment and directly induces multiple early lytic genes, is questionable. Rather, the observed phenotypes may be due to general effects on the cell via USP7 and other interactions of vIRF-2 and vIRF-3, resulting in an intracellular environment ill supportive of virus replication. The induction of innate immune signaling, such as that mediated by vIRF-2-activated TRAFs 3 and 6, would be expected to provide such nonoptimal conditions, leading to suppressed virus replication. That said, it also is apparent that vIRF-2-induced IFIT 1, 2, and 3 genes, when overexpressed in isolation, inhibit HHV-8 early lytic gene expression during *de novo* and/or lytic infection of huARLT cells, indicating a more direct suppression of lytic gene expression (39). Establishing the precise mechanisms underlying the overall effects of vIRF-2 and vIRF-3 on HHV-8 productive replication must await further detailed investigations.

An unexpected finding from our studies is that USP7 catalytic domain residues are required for the interaction of vIRF-2, but not vIRF-1, vIRF-3, and vIRF-4, with USP7 intracellularly or in the presence of other cellular proteins (i.e., cell extract). Interestingly, recombinant vIRF-2 peptide (residues 241 to 260) and USP7 NTD did interact *in vitro*. Possible explanations for these findings are that binding of vIRF-2 to USP7, in the absence of catalytic domain residues, is hindered by one or more cellular proteins, that vIRF-2-USP7 interaction affinity is enhanced by conformational changes of NTD affected by residues of the catalytic domain, and/or that one or more catalytic domain-binding cellular proteins allow stearic accessibility of vIRF-2 to the N-terminal domain of USP7. It is also possible that posttranslational modification of USP7, effected by one or more proteins in intact cells and cell lysates, promotes NTD accessibility by vIRF-2 and/or its binding affinity. Experimental exploration of the underlying basis for the unusual USP7-interacting properties of vIRF-2 could conceivably uncover novel determinants of USP7-protein interactions and interaction specificities that apply to cellular proteins as well as to vIRF-2.

Another significant finding from this study is that TRAF3, in addition to the previously reported TRAF6 (34), is an apparent substrate of USP7, as TRAF3 polyubiquitination was suppressed in the presence of overexpressed USP7. Furthermore, ubiquitin modifications of other TRAFs (1, 2, 4, and 5) reported to interact with USP7 (31, 33) were completely refractory to the effects of overexpressed USP7 in parallel transfection-based analyses, although each TRAF did interact with the deubiquitinase in this context (Fig. 5A and 6A). To our knowledge, this is the first evidence of TRAF specificity with respect to USP7 catalytic activity.

To conclude, our present studies have identified and characterized HHV-8 vIRF-2 interaction with USP7, USP7 binding-dependent enhancement by vIRF-2 of TRAF3 and TRAF6 polyubiquitination and signaling, and activities of vIRF-2 and vIRF-2-USP7 interaction in HHV-8 latent and lytic biology. The specific identification of TRAF3 and TRAF6 regulation via vIRF-2 targeting of USP7 represents a novel mechanism of viral protein activity via USP7 interaction. Overall, our findings extend the understanding of USP7-protein interactions and the functional consequences and associated mechanistic underpinnings of USP7 targeting by HHV-8 and other viral proteins.

## MATERIALS AND METHODS

### Plasmids and primers.

Plasmids encoding Flag-TRAF6 and V5-MAVS (pICE-V5-MAVS) in addition to reporter plasmid pIFN-β-Luc have been reported previously (53–55) and were provided by E. W. Harhaj and K. Tolba. Expression vectors pRK5-HA-Ubiquitin-WT and pRK5-HA-Ubiquitin-K63, encoding HA-tagged wild-type and K63-only ubiquitin (56), were obtained from Addgene (plasmid numbers 17608 and 17606) and were deposited by T. Dawson; Addgene plasmids pQHA-USP7 (46753) and pQFlag-USP7 (46751) were reported (57) and deposited by G. Peters; lentiviral packaging vectors psPAX2 and pMD2.G were obtained from Addgene (12260 and 12259, respectively; deposited by D. Trono). The reporter pRL-Luc was obtained from Promega (catalogue number E2231). Expression plasmids pCEBZ-vIRF1-SF, pCEBZ-vIRF3-SF, pCEBZ-USP7-CBD, and pET22b-USP7-NTD were reported by us previously (27). Lentiviral plasmid vector pCEBZ-vIRF2-SF (encoding StrepII- and Flag-tagged vIRF-2) was generated by insertion of PCR-generated, NotI- and BamHI-flanked vIRF-2 coding sequences into the corresponding cloning sites of pCEBZ-RFP-SF (58) (replacing RFP coding sequences). Various vIRF-2 open reading frame (ORF) fragments (Δ1 [codons 1 to 164], Δ2 [1 to 347], Δ3 [348 to 680], Δ4 [1 to 190], Δ5 [1 to 230], Δ6 [1 to 270], and Δ7 [1 to 310]), deletions or point mutations of vIRF-2 ORF sequences [Δ(241-260), m1 (alanine substitutions of codons 241 to 245), m2 (alanines 246 to 250), m3 (alanines 251 to 255), m4 (alanines 256 to 260), SP_242_AA, RP_244_AA, ST_246_AA, SQ_248_AA, VQ_250_AA, P_244_G, S_247_A, and Q_248_A] were amplified by PCR or overlapping PCR and inserted between the NotI and BamHI cloning sites of pCEBZ-RFP-SF (replacing the RFP ORF) to provide vectors expressing the respective StrepII- plus Flag-tagged proteins. Lentiviral plasmid vector pCEBZ-vIRF2-CBD and pCEBZ-vIRF4-CBD were generated by insertion of PCR-generated, NotI- and BamHI-flanked vIRF2 and vIRF4 (genomic) coding sequences between the corresponding cloning sites of pCEBZ-RFP-CBD (59). A bacterial expression vector for His_6_-tagged residues 1 to 220 of USP7 was generated by cloning of the respective USP7 ORF sequences into pET28b(+) (catalogue number 69865; Novagen). Lentiviral plasmid-based expression vectors for C-terminally CBD-tagged vIRF-2, vIRF-2.S_247_A, and USP7 fragments containing residues 1 to 207 and 1 to 220 were generated by PCR amplification of the corresponding coding sequences as NotI-BamHI fragments and their cloning between these sites in pCEBZ-RFP-CBD. Sequences encoding vIRF-2 residues 226 to 275, 226 to 245, 241 to 260, and 256 to 275 were PCR amplified and cloned into BamHI-EcoRI sites of the vector pGEX-4T-1 (catalogue number 28-9545-49; GE Healthcare) to enable bacterial expression of the N-terminally GST-fused sequences. Lentiviral plasmid vectors pCEBZ-TRAF1-S, pCEBZ-TRAF2-S, pCEBZ-TRAF3-S, pCEBZ-TRAF4-S, and pCEBZ-TRAF5-S were generated by insertion of PCR-generated, NotI- and BamHI-flanked TRAF coding sequences into the corresponding cloning sites of pCEBZ-RFP-S (59). For vIRF-2 depletion, mRNA-directed shRNAs were cloned between the BamHI and MluI sites of pYNC352 lentiviral vector (60); target sequences were GGAGCGACATAATCGAGAA (shRNA 1), GGTTTACCTTCTCCTGTTA (shRNA 2), and GGTGTTTGTTAGATCTCTT (shRNA 3). To generate shRNA-resistant vectors expressing vIRF-2 and vIRF-2.S_247_A, codon-synonymous mutations within the shRNA 3 target sequence were introduced into the respective vIRF-2 ORFs by overlapping PCR-mediated mutagenesis; the shRNA-resistant coding sequences were cloned between the NotI and BamHI sites of pCEBZ-CBD.

### HHV-8 mutagenesis.

Mutagenesis of HHV-8 bacmid BAC16 (61) for generation of HHV-8 vIRF-2-null (ATG to TTG) and vIRF-2.S_247_A mutants and revertants thereof was essentially as described previously (27, 49, 62). Forward and reverse primers for ATG mutagenesis were 5′-acggaaaaggtgttttgtgtcgtggcttttgcctaaaaagTtgcctcgctacacggagtcaggatgacgacgataagtaggg-3′ (uppercase letter, introduced mutation; underlined, homologous to Kan^r^-flanking sequence) and 5′-ataaagtccgtgagccattccgactccgtgtagcgaggcaActttttaggcaaaagccaccaaccaattaaccaattctgattag-3′. Equivalent primers containing wild-type sequences were used for reversion of this mutation to generate the repaired control virus. Forward and reverse primers used for S_247_A mutagenesis of vIRF-2 were 5′-tcgagcttattagctccggattccccgcgtccctccacgGCCcaggtgcagggcccattacaaggatgacgacgataagtaggg-3′ and 5′-atccgtcggggtgtgcacgtgtaatgggccctgcacctgGGCcgtggagggacgcggggaatcaaccaattaaccaattctgattag-3′. Equivalent primers containing wild-type sequences were used for reversion of this mutation to generate the repaired control virus. Introduced mutations were verified by sequencing, and overall integrities of engineered genomes were checked by restriction analysis.

### Cell culture and transfection.

TRExBCBL1-RTA (38) and JSC-1 (63) cells were grown in RPMI 1640 medium supplemented with 10% fetal bovine serum (FBS) and 10 μg/ml gentamicin. HEK293T and iSLK cells (50) were cultured in Dulbecco’s modified Eagle’s medium (DMEM) supplemented with 10% FBS and 10 μg/ml gentamicin. HEK293T cells cultured to 40 to 60% confluence were transfected with mixtures of plasmid DNA and cationic polymer linear polyethylenimine (593215; Polysciences).

### Immuno- and affinity precipitations.

For Flag-based immunoprecipitation and CBD- and S-tag-based affinity precipitations for assessments of protein-protein interactions, HEK293T cells in 10-cm dishes were cotransfected with appropriate bait and prey expression vectors. At 48 h posttransfection, cells were harvested and lysed for 1 h at 4°C in 0.2% NP-40 lysis buffer (50 mM Tris-HCl [pH 7.5], 150 mM NaCl, 5 mM EDTA, and 0.2% NP-40) containing protease inhibitor cocktail (P8340; Sigma). Lysates were subsequently clarified by centrifugation at 16,000 × *g* for 10 min at 4°C. The supernatants were used in the subsequent coprecipitation assays, employing Flag antibody beads (catalogue number M8823; Sigma), chitin resin (S6651; New England Biolabs), or S-protein agarose (69704; Novagen); these were incubated with lysates at 4°C overnight with gentle shaking. Precipitates were then washed 3 to 5 times with wash buffer (50 mM Tris-HCl [pH 7.5], 150 mM NaCl, and 0.05% NP-40). Coprecipitated proteins were released from the beads by incubation at 95°C for 10 min in gel loading buffer (100 mM Tris [pH 6.8], 4% SDS, 200 mM dithiothreitol, 0.2% bromophenol blue, 20% glycerol) prior to polyacrylamide gel electrophoresis and immunoblotting. For preparation of denatured cell extracts for analyses of TRAF ubiquitination, cells were treated with 150 μl of PBS (137 mM NaCl, 2.7 mM KCl, 10 mM Na_2_HPO_4_ [pH 7.4]) containing 1% SDS and boiled for 10 min. Subsequently, lysates were diluted 1:3 with PBS and sonicated using a microtip sonicator (5 min per sample). After centrifugation at 16,000 × *g* for 10 min at 4°C, the supernatants were harvested and diluted 5-fold in PBS. Supernatants were incubated with Flag antibody beads (catalogue number M8823; Sigma) or S-protein agarose (catalogue number 69704; Novagen) at 4°C overnight, and then beads and associated proteins were pelleted by microcentrifugation or magnetic capture (Flag) and washed in lysis buffer by repeated resuspension and pelleting prior to protein release by heating/denaturation (S-protein agarose) or application of 100 ng/ml of 3× Flag peptide (catalogue number F4799; Sigma) in lysis buffer (100 μl, ×2) and analysis of released proteins by SDS-PAGE and immunoblotting. For cross-linking-based immunoprecipitation assays (to detect endogenous TRAF-USP7 and vIRF-2-USP7 interactions), cells were washed twice with PBS to remove media and then were incubated with disthiobis-succinimidyl propionate (DSP) cross-linker solution (1 to 2 mM DSP in PBS). After 30 min of incubation at room temperature, the cells were incubated with stop solution (10 to 20 mM Tris-HCl [pH 7.4] final concentration) for 15 min. Immuno- and affinity precipitations were carried out on these samples as outlined above for the denatured cell extracts. For *in vitro* coprecipitation assays, appropriate expression vectors were used to transform Escherichia coli BL21(DE3) to produce GST- or His_6_-linked proteins. After 24 h of induction at 18°C by addition of 1 mM isopropyl-β-d-thiogalactopyranoside (IPTG), the cell pellets were resuspended in lysis buffer (300 mM NaCl, 10 mM imidazole, 50 mM NaH_2_PO_4_ [pH 8.0]) for His_6_-tagged protein or PBS for GST fusions in the presence of 1 mg/ml lysozyme. After 10 min of sonication, the lysates were cleared by centrifugation at 12,000 × *g* for 10 min at 4°C. The supernatants then were incubated with either nickel-nitrilotriacetic acid (catalogue number 30210; Qiagen) or glutathione Sepharose (catalogue number 17-0756-01; GE Healthcare) beads at 4°C for 1 h. Subsequently, the beads were washed 3 to 5 times with lysis buffer (His_6_ precipitates) or PBS (GST precipitates) and proteins released with either His_6_ elution buffer (300 mM NaCl, 250 mM imidazole, 50 mM NaH_2_PO_4_ [pH 8.0]) or glutathione buffer (50 mM Tris-Cl [pH 8.0], 10 mM reduced glutathione). Purified proteins were checked for purity by Coomassie brilliant blue staining. For coprecipitation assays, GST or GST fusion proteins were incubated on ice for 2 h with His_6_-fused USP7 proteins in PBS. The samples were incubated with 30 μl of glutathione Sepharose on ice for 2 h. The beads were then collected and washed 5 times with ice-cold PBS. Finally, the proteins were eluted with glutathione buffer and then boiled for 10 min in SDS-PAGE loading buffer prior to gel electrophoresis and immunoblotting (for detection of GST and His_6_) or Ponceau S staining for global protein detection.

### Antibodies.

The following primary antibodies were used: S-tag (catalogue number ab184223; Abcam); CBD (E8034S; New England BioLabs); Flag (F1804; Sigma); β-actin (A5316; Sigma); LANA (13-210-100; Advanced Biotechnologies); USP7 (A300-033A [rat]; Bethyl Laboratories); V5-tag (R960-25; Thermo Fisher Scientific); and LDH, HA, His_6_, GST, and p53 from Santa Cruz Biotechnologies (catalogue numbers sc-33781, sc-7392, sc-803, sc-138, and sc-126, respectively). vIL-6 rabbit polyclonal antiserum was reported previously (64). Mouse monoclonal antibody to vIRF-2 (39) was provided by T. Schulz. Horse radish peroxidase (HRP)-conjugated anti-rabbit IgG and anti-mouse IgG were from Cell Signaling Technology (catalogue numbers 7074S and 7076S, respectively).

### Production of lentiviruses and HHV-8 (BAC16).

For lentivirus production, ∼80% confluence HEK293T cultures in T75 flasks were cotransfected with 12 μg of lentiviral vector and 9 μg and 3 μg, respectively, of psPAX2 and pMD2.G packaging vectors. Culture media were replaced by fresh 10% FBS-containing Dulbecco’s modified Eagle’s medium (DMEM) after 6 h, and 48 h later, the media were harvested and lentiviruses pelleted by ultracentrifugation at 25,000 rpm using an SW-32 Ti rotor for 2 h at 4°C. Viral pellets were resuspended in 5 ml RPMI 1640 medium containing 10% FBS; aliquots were stored at –80°C. For HHV-8 BAC16 virus production, BAC16 DNA was transfected into iSLK cells with FuGENE 6 transfection reagent (catalogue number E2693; Promega), and BAC16-containing cells (green fluorescent protein [GFP] positive) were selected by treatment of cultures with 2.3 mM hygromycin B (catalogue number 10687010; Invitrogen) for 3 weeks. Productive replication was induced in these cultures by addition of doxycycline (1.9 nM) and sodium butyrate (1 mM). Virus-containing media were harvested 3 days and 5 days postlytic induction, with medium replacement after 3 days, and the two medium sets were combined. Virus was pelleted by ultracentrifugation in an SW-32 Ti rotor at 25,000 rpm for 2 h at 4°C; viral pellets were resuspended in 5 ml DMEM supplemented with 10% FBS, and aliquots of virus were stored at –80°C.

### Measurements of cell growth and apoptosis.

To assess the effects of vIRF-2 on cell growth and viability, TRExBCBL1-RTA and JSC-1 cells were transduced with nonsilencing (NS) control or vIRF-2 mRNA-directed shRNA-encoding lentivirus at a multiplicity of infection yielding a rate of transduction of ∼90% (determined by the proportion of cells positive for GFP encoded by the lentiviral vector pYNC352) by incubation of cells with lentivirus in the presence of 5 μg/ml Polybrene for 6 h, prior to replacement of lentivirus medium with fresh medium. Cells were allowed to rest for 2 to 3 days prior to cell density normalization and experimental use. For growth assays, cells were seeded into 24-well tissue culture plates, and hemocytometric counting of trypan blue-excluding (viable) cells was carried out daily for 3 days. For each experiment, triplicate cultures were analyzed. To assess apoptosis, cells from the last day of growth experiments were collected and washed with annexin V binding buffer (10 mM HEPES [pH 7.4], 140 mM NaCl, and 2.5 mM CaCl_2_), and annexin V-Cy3 reagent (BioVision, catalogue number 1002-200) was added to the cells at a dilution of 1:500 in annexin V binding buffer for 5 min in the dark. The cells were then washed twice by suspension in binding buffer and pelleting by centrifugation prior to cell fixing and nuclear staining by incubation in the dark for 15 min in PBS containing 4% paraformaldehyde (PFA) and Hoechst 33342 stain (1 μg/ml). Cells were washed in PBS, resuspended in PBS containing 4% PFA, and transferred to a microscope slide; after desiccation, cells were overlaid with 90% glycerol and a coverslip for visualization of Cy3 (annexin V, red) and Hoechst (nuclei, blue) fluorescence. Four random fields were analyzed for each sample, with >900 cells evaluated for calculation of the percentage of apoptotic cells.

### HHV-8 replication assays.

For assessment of productive replication in TRExBCBL1-RTA PEL cells, cultures were treated with doxycycline (1 μg/ml) for 24 h to induce lytic reactivation and incubated for a further 3 days. Virus-containing media were harvested for measurements of virus yield. Infectious virus titers were determined by immunofluorescence assay (IFA) for LANA expression in diluted medium-inoculated iSLK cells. At 24 h postinfection, cells were washed twice with PBS, fixed with 4% PFA for 20 min, and permeabilized with 0.25% Triton X-100 in PBS for 5 min, prior to blocking with PBS containing 2% bovine serum albumin (BSA) and 5% normal goat serum (NGS) for 2 h. LANA antibody was added at a dilution of 1:1,000 for overnight incubation at 4°C, and following three rinses with washing buffer (3% Triton X-100 in PBS), Cy3-conjugated secondary antibody was added at a dilution of 1:400 for 1 h at room temperature. Following 5 washes with PBS, nuclei then were stained using Hoechst 33342 (described above) and cells were visualized by UV microscopy. Random fields were visualized to calculate the percentage of LANA-positive cells (at least 1,000 cells were assessed per sample). For determinations of encapsidated viral genome yields, qPCR was used. Following pretreatment of virus suspensions with DNase I overnight at 37°C to remove any unencapsidated viral DNA, viral DNA was extracted using silica-guanidinium thiocyanate. Briefly, to 300 μl DNase I-treated medium sample was added 1 ml of 6 M guanidine thiocyanate containing 5 μg of silica gel (catalogue number S5631; Sigma), the mixture incubated at 55°C for 10 min, and matrix-bound DNA harvested and washed by centrifugation and resuspension in PBS; DNA was released from the silica gel by addition of distilled water. Quantitative PCRs, employing ORF73 sequence-directed primers, were performed in a 96-well microplate with SYBR green 2× master mix as described previously (15). For assessments of wild-type and mutant BAC16 virus replication in iSLK cells, iSLK cultures at ~30% confluence were infected with equal infectious doses of viruses, and BAC16-containing cells were selected by hygromycin treatment (as described above for virus production). Virus lytic replication was induced with doxycycline (1.9 nM) and sodium butyrate (1 mM), and virus-containing media were harvested 3, 6, and 9 days after lytic induction and combined for assays of infectious virus and encapsidated viral genome titers (described above).

### RT-qPCR.

RNA was isolated from cells using TRIzol reagent (catalogue number 15596026; Invitrogen) according to the manufacturer’s protocol. One microgram of total RNA was treated with 1 U of RNase-free DNase I (catalogue number M0303S; New England BioLabs) for 30 min at 37°C prior to heat inactivation of the enzyme at 65°C for 10 min. Reverse transcription (RT) was performed using 200 U of Superscript III reverse transcriptase (catalogue number 56575; Invitrogen) in the presence 2.5 μM random hexamer deoxyoligonucleotides and 0.5 mM deoxynucleotide triphosphates in RT buffer (50 mM Tris-HCl [pH 8.3], 75 mM KCl, 3 mM MgCl_2_). First-strand cDNA then was analyzed by quantitative PCR using Power SYBR green PCR master mix (catalogue number 4367659; Thermo Fisher Scientific), as described previously (15).

### Luciferase reporter assay.

Reporter assays utilized a reporter plasmid, IFN-β-Luc (55), comprising the IFN-β promoter driving firefly luciferase ORF expression. This was transfected into HEK293T cells along with pRL-TK (catalogue number E2241; Promega), for constitutive expression of Renilla luciferase (providing a normalization control), using 50 ng and 5 ng of the respective plasmids per well of a 12-well tissue culture plate; cotransfected plasmids included expression vectors for MAVS (25 ng), USP7 (425 ng), and/or vIRF-2 (425 ng). After 24 h, cells were lysed and analyzed for luminescence using Dual-Glo reagent (catalogue number E2920; Promega) and a Promega GloMax multidetection system.
